# Predicting skin melanoma progression via LAG-3, TIGIT and HAVCR2

**DOI:** 10.1007/s10142-026-01832-0

**Published:** 2026-02-26

**Authors:** Paraskevi Vryza, Ilias Georgakopoulos-Soares, Apostolos Zaravinos

**Affiliations:** 1 Cancer Genetics, Genomics and Systems Biology Laboratory, Basic and Translational Cancer Research Center (BTCRC), Nicosia, 1516 Cyprus; 2 Department of Life Sciences, School of Sciences, European University Cyprus, Nicosia, 1516 Cyprus; 3 Division of Pharmacology and Toxicology, College of Pharmacy, Dell Paediatric Research Institute, The University of Texas at Austin, Austin, TX USA

**Keywords:** LAG3, TIGIT, HAVCR2, Skin cutaneous melanoma, Immune checkpoints, Prognostic biomarkers, Gene expression

## Abstract

Skin cutaneous melanoma (SKCM) is a highly aggressive malignancy with increasing global incidence and mortality. While immune checkpoint inhibitors, predominantly PD-1/PD-L1 blockers, have improved outcomes, resistance and immune-related toxicity remain major challenges. Relatlimab, a lymphocyte activation gene-3 (LAG3) inhibitor, in combination with PD-1 inhibitors, has shown clinical promise, while emerging immune regulators such as *TIGIT* and *HAVCR2* are being investigated as potential immunotherapeutic targets. Understanding their roles in SKCM could provide novel therapeutic insights. We investigated the emerging checkpoints LAG3, TIGIT, and HAVCR2 in SKCM using in silico analyses. We analyzed gene expression, immune infiltration, mutational profiles, and methylation patterns using bioinformatic tools, including TIMER, CCLE, Maftools, and UALCAN. Protein expression data were obtained from the Human Protein Atlas, and survival analyses were conducted using Kaplan–Meier plots and Cox regression. Additional molecular and prognostic assessments were performed using MethSurv, Sangerbox, and TISIDB. *LAG3*, *TIGIT*, and *HAVCR2* were significantly upregulated in SKCM, with higher expression in metastatic compared with primary tumors and associated with improved cumulative survival. Their high expression correlated with increased immune infiltration, particularly by T cells and macrophages, and higher ImmuneScore and StromalScore. Epigenetic regulation through promoter hypermethylation was associated with reduced expression of these genes, suggesting potential immune modulatory mechanisms. Our findings indicate that *LAG3*, *TIGIT*, and *HAVCR2* may serve as prognostic biomarkers and immunotherapeutic targets in skin melanoma, warranting further investigation. Their roles in immune regulation and tumor progression underscore their relevance in shaping the tumor microenvironment. Future studies should explore their therapeutic potential, particularly in combination with existing immune checkpoint inhibitors, to enhance treatment efficacy. High expression was associated with improved survival; after adjustment for age, AJCC staging, ImmuneScore, and StromalScore, LAG3 and TIGIT showed independent prognostic value, whereas HAVCR2 did not remain significant after FDR correction. Immune-stratified analyses showed that prognostic associations were strongest in immune-high tumors.

**Supplementary Information:**

The online version contains supplementary material available at 10.1007/s10142-026-01832-0.

## Introduction

Skin cutaneous melanoma (SKCM) is an aggressive form of cancer with a high rate of metastasis and a poor prognosis for advanced-stage patients (Arnold et al. 2022; Didier et al. 2024). It accounts for ~ 2% of the global cancer burden while it is responsible for 80% of skin malignancy-related deaths, with an increasing incidence, especially among Caucasian populations (Anagnostou et al. 2020; Martín-Lluesma et al. 2024). Cutaneous melanoma is typically driven by environmental influences, epigenetic effects, and genetic predisposition (Caini et al. 2009). Ultraviolet (UV) radiation from sunlight or artificial sources is the primary environmental risk factor. Familial cases encompass ~ 10% of melanoma cases (Arnaut et al. 2021). The recurrently mutated genes in the disease have been well-established, and include *BRAF*,* NRAS*,* MITF*,* KIT*,* TP53*,* CDKN2A* and *PTEN*, as well as newly discovered ones, such as *NF1*,* RAC1*,* IDH1* and *ARID2*. Notably, *BRAF* mutations, which drive uncontrolled MAPK pathway activation, are found in ~ 40–60% of the cases, while *NRAS* mutations occur in ~ 15–30% of the cases, often in a mutually exclusive manner (Ottaviano et al. 2021; Colombino et al. 2012; Hayward et al. 2017).

Primary melanoma management typically includes wide local excision with margins determined by tumor thickness (Breslow score thickness), alongside surgical procedures, radiation therapy, targeted therapy and immunotherapy (Long et al. 2023). Importantly, over the past decade, immune checkpoint inhibition (ICI) therapies targeting cytotoxic T-lymphocyte-associated antigen-4 (CTLA-4) (e.g., Ipilimumab or Tremelimumab) and programmed cell death protein 1 (PD-1) (e.g., Nivolumab, Pembrolizumab, Cemiplimab, Dostarlimab, Retifanlimab and Toripalimab) have significantly improved melanoma treatment outcomes, demonstrating durable responses (Long et al. 2023). In 2011, the FDA approved anti-CTLA-4 monoclonal antibodies for stage IV melanoma treatment, followed by the approval of anti-PD-1 inhibitors as second-line treatments for metastatic disease. Nevertheless, PD-1/PD-L1 and CTLA-4 inhibitors exhibit varying degrees of drug resistance and immune-related toxicities, highlighting the need for alternative immunotherapeutic targets (Martín-Lluesma et al. 2024; Das et al. 2015; Seth et al. 2020; Bagchi et al. 2021; Chauvin and Zarour 2020; Koyama et al. 2016; Gide et al. 2018).

Lymphocyte activation gene 3 (LAG3), T cell immunoreceptor with immunoglobulin and ITIM domain (TIGIT), and hepatitis A virus cellular receptor 2 (HAVCR2/TIM-3) are emerging immune checkpoint molecules associated with T-cell exhaustion and immune evasion in melanoma. LAG3, located on chromosome 12p13.31, encodes an inhibitory receptor that negatively regulates T-cell activation and enhances regulatory T-cell (Treg) function. Due to its immunosuppressive role, LAG3 has become a promising immunotherapy target, with Relatlimab—the first anti-LAG3 monoclonal antibody—gaining FDA approval in combination with Nivolumab for the treatment of advanced melanoma (Long et al. 2023; Das et al. 2015).

HAVCR2, located on chromosome 5q33.2, encodes TIM-3, a negative regulator expressed on Th1, CD8 + T, and Treg cells, where it suppresses immune responses via interactions with galectin-9. TIM-3 is upregulated in several malignancies, including melanoma, non-small cell lung cancer (NSCLC), and hepatocellular carcinoma, making it an important immunotherapeutic target (Thorsson et al. 2018; Pavelescu et al. 2024; Li et al. 2022).

Similarly, TIGIT, encoded on chromosome 3q13.31, is a co-inhibitory receptor that suppresses T and NK cell function, promoting Treg activity, and modulating dendritic cells. TIGIT expression is associated with tumor immune evasion in melanoma, and its blockade has demonstrated potential in restoring antitumor immunity (Bagchi et al. 2021). This result underscores TIGIT’s role in tumor immune evasion and its utility in dual checkpoint blockage; combining TIGIT with PD-1/PD-L1 inhibitors (Dummer et al. 2025). 

Despite advances in immunotherapy, clinical benefits remain limited in certain patient populations, underscoring the need for new therapeutic approaches. This study aims to evaluate the roles of LAG3, TIGIT and HAVCR2 in skin melanoma by analyzing their diagnostic and prognostic significance, their immune infiltration patterns, protein interactions, genomic alterations, and methylation profiles. Understanding the molecular and functional roles of these emerging immune checkpoints may contribute to the development of improved immunotherapeutic strategies for the disease.

## Methods

### Protein expression

The Human Protein Atlas (HPA) (https://www.proteinatlas.org/) was used to assess protein expression in SKCM and normal tissues (Rozanova et al. 2021; Digre and Lindskog 2021).

### Immune cell infiltration

The Tumor Immune Estimation Resource (TIMER) was used to analyze immune cell infiltration in SKCM and perform gene expression correlations (Ma and Zhou 2022).

### CCLE

The Cancer Cell Line Encyclopedia (CCLE) is a comprehensive repository that provides gene expression, mutation, and drug response data from a wide array of cancer cell lines. We used CCLE to investigate the expression of LAG3, TIGIT and HAVCR2 across multiple tumor cell lines, allowing for the assessment of their molecular characteristics in different cancer subtypes (Nusinow et al. 2020).

### Sangerbox

Sangerbox (http://sangerbox.com) was used to explore the association between *LAG3*,* TIGIT* and *HAVCR2* and five distinct categories of marker genes within the SKCM immune pathway, encompassing immune checkpoints (Thorsson et al. 2018) and a set of 150 immunomodulators. Additionally, we systematically analyzed datasets from the TCGA database, incorporating cancer stemness indices and genetic mutations (Chen et al. 2024).

### UALCAN

The University of ALabama at Birmingham CANcer data analysis Portal (UALCAN) (http://ualcan.path.uab.edu) provides pre-computed analyses of tumor subgroup-specific promoter methylation status. We used it to examine the promoter methylation status of *LAG*−3, *TIGIT* and *HAVCR2* in SKCM. Furthermore, the MethSurv database was used to assess the prognostic significance of methylation levels for these genes through multivariable survival analysis (Han et al. 2021).

### cBioPortal

The cBio Cancer Genomics Portal (http://cbioportal.org) is a web-based platform designed to interactively explore diverse cancer genomic datasets (Feng et al. 2022). We utilized this tool to examine the expression of *LAG*3, *TIGIT* and *HAVCR2* across distinct CNV groups associated with these genes.

### Maftools

We used Maftools (Mayakonda et al. 2018) to investigate mutations affecting *LAG3*, *TIGIT* and *HAVCR2* across skin melanomas in the TCGA-SKCM dataset, according to the top recurrently mutated genes.

### MicroRNA interaction

We explored the list of miRNAs interacting with *LAG*3, *TIGIT*, and *HAVCR3* utilizing miRWalk (http://mirwalk.umm.uni-heidelberg.de/), miRDB (http://mirdb.org) (Chen and Wang 2020)and miRabel (Quillet et al. 2020). To assess the differential expression, prognostic implications, and diagnostic potential of these target miRNAs in skin melanoma, we used the CancerMIRNome tool (https://ngdc.cncb.ac.cn/databasecommons/database/id/8011). Multiple entities for the same miRNA predicted binding sites within the target transcript.

### Survival analysis

The Kaplan-Meier plotter (http://kmplot.com) was used to assess the prognostic impact of *LAG3*,* TIGIT*, and *HAVCR2* in skin melanoma. The genes’ influence on immunotherapy was investigated concerning overall survival (OS) with both anti-PD-1 treatment and Nivolumab-only treatment. The analysis established Hazard Ratios (HR), 95% Confidence Intervals (CI), and logarithmic p-values to delineate their significance.

### TISIDB

Tumor and Immune System Interaction Database (TISIDB, http://cis.hku.hk/TISIDB/) was utilized to analyze the expression of *LAG*3, *TIGIT* and *HAVCR2* concerning their immune subtypes (C1-C6), as proposed by Thorsson et al. (Thorsson et al. 2018), as well as the molecular subtypes in skin melanoma (BRAF mutants, NF1 mutants, RAS mutants and Triple-WT).

### Statistical analysis

Statistical significance was determined using unpaired Wilcoxon Rank Sum tests. Survival analysis was conducted using Kaplan-Meier plots and Cox regression models. A *p* < 0.05 was used as a threshold for statistical significance. All analyses involving multiple comparisons were corrected using the Benjamini–Hochberg false discovery rate (FDR). Effect sizes (log2 fold change, Spearman’s rho, hazard ratios, and regression coefficients) are reported alongside p and FDR-adjusted values.

To evaluate whether the prognostic effects of *LAG3*, *TIGIT* and *HAVCR2* were independent of immune infiltration, ImmuneScore and Stromal Score were calculated using the ESTIMATE algorithm on TCGA-SKCM RNA-seq data. We combined these scores with curated clinical variables (overall survival, status, age, stage) using TCGA sample identifiers. Gene expression values were derived from STAR-aligned raw counts, normalized using the edgeR pipeline, and transformed to log-CPM.

Multivariable Cox regression models were performed in R (version 4.5.1) using the survival package, with each gene (continuous variable) and ImmuneScore, Stromal Score, age and stage included as covariates where available. Hazard ratios (HR), 95% confidence intervals (CI), and p-values were reported, and Benjamini–Hochberg FDR correction was applied to adjust for multiple testing.

Since immune infiltration may modify the prognostic implications of immune-checkpoint gene expression, we additionally stratified Cox proportional hazards models in subgroups defined by high vs. low ImmuneScore (median split). For each subgroup, the association of *LAG3*, *TIGIT* and *HAVCR2* expression with overall survival was estimated separately. The same modelling framework was applied, and raw p-values were reported.

## Results

### Differential expression of *LAG3*, *TIGIT* and *HAVCR2*

We first analyzed the expression of *LAG3*, *TIGIT* and *HAVCR2* across multiple tumor types in the TCGA TARGET GTEx pan-cancer dataset (PANCAN, *n* = 19,131) and focused on skin melanoma. Expression values were log_2_(x + 0.001) transformed and compared between tumor and normal tissues using the Wilcoxon test, excluding cancer types with fewer than three samples.

**Fig. 1:**
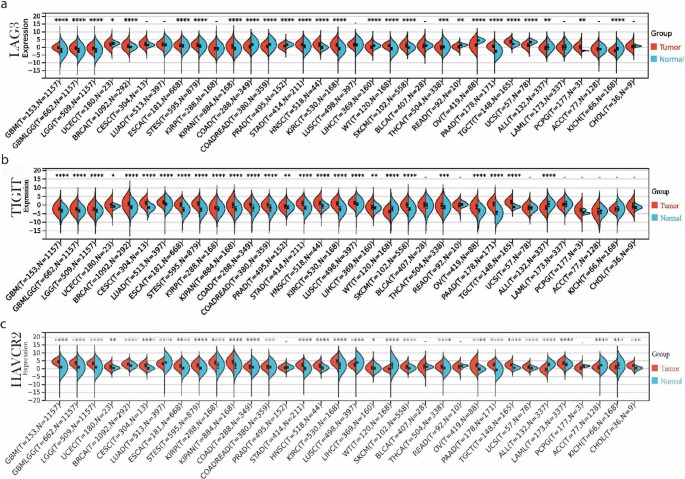
Expression and prognostic significance of *LAG3* (**a**), *TIGIT* (**b**) and *HAVCR2* (**c**) in skin melanoma, in pan-cancer and cell lines. Gene expression was measured across 34 tumor types based on TCGA and GTEx datasets. *, *p* ≤ 0.05; **, *p* ≤ 0.01; ***, *p* ≤ 0.001; ****, *p* ≤ 0.0001. Unless otherwise noted, all p values are FDR-adjusted

*LAG3* was significantly upregulated in most of the tumor types analyzed, including glioblastoma multiforme (GBMT), low-grade glioma (LGG), breast cancer (BRCA), esophageal cancer (ESCA), stomach and esophageal carcinoma (STES), kidney cancer (KIPAN), stomach adenocarcinoma (STAD), head and neck squamous cell carcinoma (HNSC), kidney renal clear cell carcinoma (KIRC), pancreatic adenocarcinoma (PAAD), testicular germ cell tumors (TGCT), pheochromocytoma and paraganglioma (PCPG) and skin melanoma (SKCM). On the other hand, it was downregulated in colon adenocarcinoma (COAD), rectum adenocarcinoma (READ), liver hepatocellular carcinoma (LIHC), ovarian serous cystadenocarcinoma (OV), uterine carcinosarcoma (UCS), acute lymphocytic leukemia (ALL) and kidney chromophobe carcinoma (KICH) (Fig. 1a). *TIGIT* and *HAVCR2* exhibited a similar expression pattern, being significantly upregulated in 24 tumor types. Just like *LAG3*,* TIGIT* was significantly elevated in skin melanoma compared to the normal skin (expression ± SD; − 1.18 ± 1.86 vs. − 1.71 ± 1.23; *p* = 3.4 × 10⁻³) (Fig. 1b), as was *HAVCR2* (1.33 ± 1.37 vs. 0.35 ± 0.73; *p* = 1.1 × 10⁻¹⁴) (Fig. 1c).

In addition, *LAG3*, *TIGIT* and *HAVCR2* were significantly upregulated in metastatic skin melanomas compared to primary ones, reflecting a more immunosuppressive tumor microenvironment in the metastatic tumors, as well as T cell exhaustion in them (Figure s1).

### Immune and molecular subtyping

**Fig. 2:**
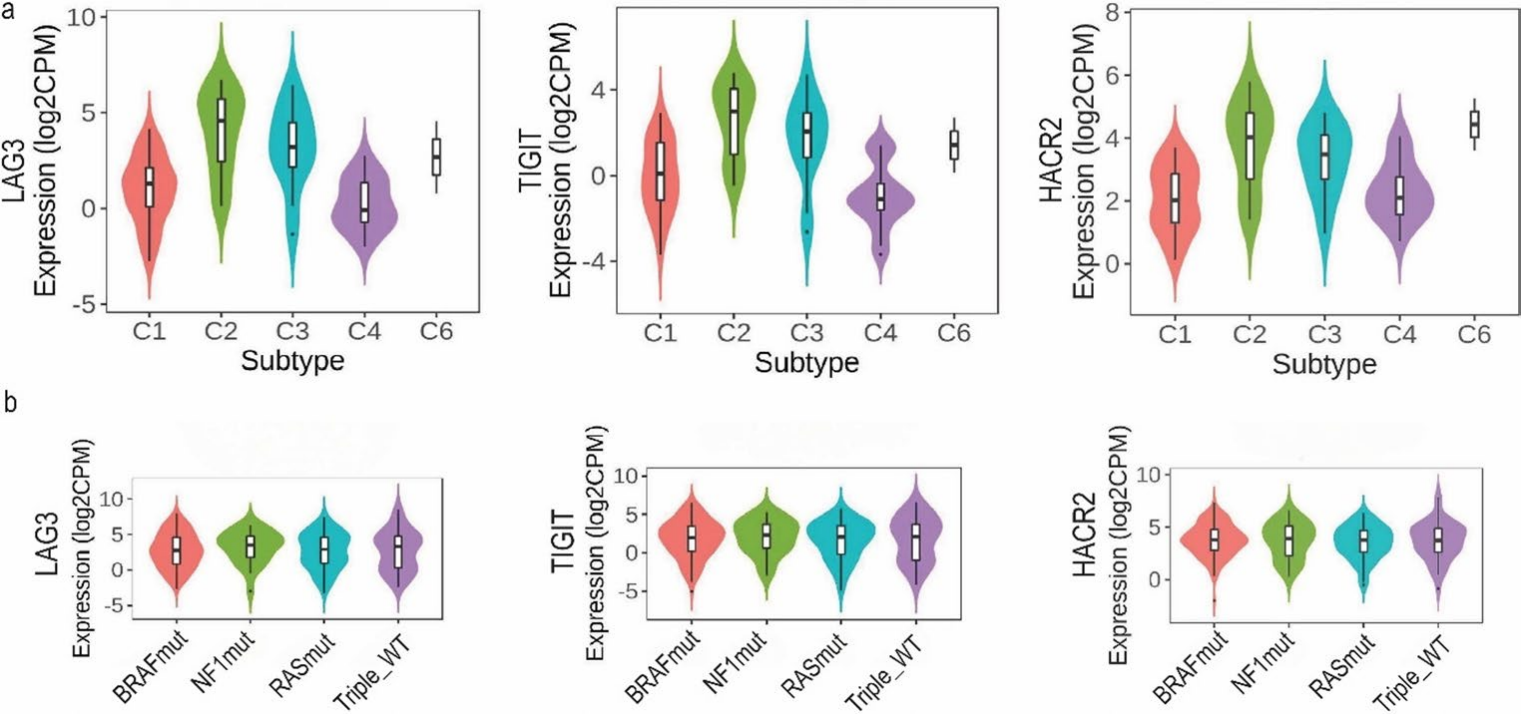
Stratification of skin melanomas by immune subtypes (C1-C6) using TISIDB indicates that *LAG3*, *TIGIT* and *HAVCR2* expression is highest in the C2 and C3 immune subtypes of skin melanoma (**a**). *LAG3*, *TIGIT* and *HAVCR2* expression across different molecular subtypes in skin melanoma (BRAF, NF1, RAS, Triple_WT) showed no significant differences between them (**b**)

Immune subtyping (C1-C6) showed that *LAG3*, *TIGIT* and *HAVCR2* were predominantly expressed in the C2 and C3 subtypes (Fig. 2a). In specific, the C2 immune subtype (also known as IFN-γ dominant) is characterized by strong T cell signals, high M1 macrophages, high PD-L1 expression, and intense immune activity; whereas, the C3 immune subtype (inflammatory) is characterized by high Th1/Th17 signaling and inflammation, but lower proliferation and better prognosis. Tumors classified as C2 or C3 have a “hot” immune microenvironment—active, but under immune control via checkpoint molecules. The over-expression of *LAG3*,* TIGIT* and *HAVCR2* in these subtypes suggests an adaptive immune resistance mechanism, as well as a rationale for immunotherapy targeting these checkpoints, especially in C2 tumors.

No significant differences in gene expression were observed across SKCM molecular subtypes for any of the three genes (Fig. 2b), clearly showing that their expression is independent of the tumor’s mutational subtype (i.e., BRAF^V600E^, NF1 mutations, NRAS/KRAS mutations or Triple^WT^) and may instead, be governed by the immune context or the microenvironment, not by the driver mutation. This suggests that immunotherapy targeting *LAG3*,* TIGIT* and *HAVCR2* can be broadly applicable across all molecular subtypes of melanoma, and not limited to BRAF-mutant, NRAS-mutant or NF1-mutant tumors.

### Clinical relevance and prognostic value of *LAG3*, *TIGIT* and *HAVCR2* in skin melanoma

**Fig. 3:**
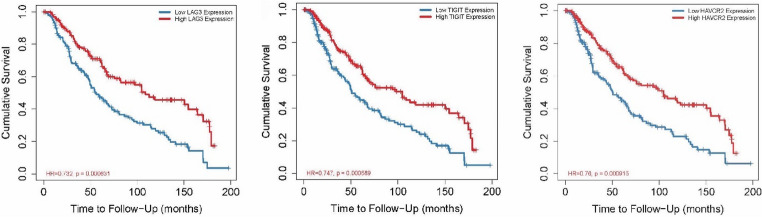
Kaplan–Meier survival analysis (TIMER 2.0, Gene_Outcome module; adjusted for age, gender, tumor purity) indicating that high *LAG3*, *TIGIT* and *HAVCR2* expression is significantly associated with improved overall survival in primary and metastatic skin melanoma. Patients were stratified into high- and low-expression groups based on the median transcript level of each gene

We further assessed the impact of abnormal *LAG3*, *TIGIT* and *HAVCR2* expressions on the SKCM patients’ prognosis using Kaplan–Meier (KM) survival curves. We utilized the TIMER 2.0 tool and through the Gene_Outcome module we used a proportional hazard model for the evaluation of the outcome significance of gene expression, adjusted by age, gender and tumor purity. According to the KM curves plotted for each gene both in primary and metastatic skin melanomas (SKCM-M), we identified that they are all associated with a decreased risk (*p* < 0.05, z < 0) of skin melanoma and particularly the metastatic one. The outcomes indicated that *LAG3*, *TIGIT* and *HAVCR2* expression separately is a associated with improved survival in the disease. In specific, high *LAG*3 expression was significantly associated with increased cumulative survival (HR = 0.732, *p* = 0.0006). Likewise, both high *TIGIT* (HR = 0.747, *p* = 0.0006) and high *HAVCR2* expression (HR = 0.76, *p* = 0.0009) were significantly correlated with improved cumulative survival (Fig. 3).

Further Kaplan-Meier survival analysis demonstrated that low *LAG3* expression was associated with poor prognosis in both primary and metastatic skin melanomas (TCGA-SKCM: *n* = 444, *p* = 1.6e-8, HR = 0.85 [0.80–0.90]; TCGA-SKCM-M: *n* = 347, *p* = 2.7e-7, HR = 0.85 [0.80–0.91]). Disease**-**specific survival (DSS) also confirmed that high LAG3 expression is positively correlated with prognosis (TCGA-SKCM: *n* = 438, *p* = 2.5e-8, HR = 0.84 [0.79–0.89]; TCGA-SKCM-M: *n* = 341, *p* = 2.4e-7, HR = 0.84 [0.79–0.90]). Progression-free interval (PFI) analysis further identified a protective role of *LAG3* expression in primary SKCM (TCGA-SKCM-P: *n* = 97, *p* = 0.03, HR = 0.85 [0.73–0.99]). Notably, low *LAG3* expression remained significantly associated with poor prognosis in metastatic skin melanomas (TCGA-SKCM: *n* = 444, *p* = 1.6e-8, HR = 0.85 [0.80–0.90]; TCGA-SKCM-M: *n* = 347, *p* = 2.7e-7, HR = 0.85 [0.80–0.91]) (Figure s2a).

Similarly, survival analysis showed that high *TIGIT* expression is linked with enhanced overall survival in patients with metastatic melanoma (TCGA-SKCM-M: *n* = 341, *p* = 1.5e-5, HR = 0.89 [0.85–0.94]), indicating its potential as a prognostic indicator. In contrast, DSS analysis revealed that high TIGIT expression is correlated with poor prognosis in primary tumors, suggesting possible context-dependent roles. PFI outcomes further supported the prognostic relevance of *TIGIT*, where reduced expression levels were significantly associated with poor prognosis both in primary and metastatic skin melanomas (TCGA-SKCM: *n* = 434, *p* = 1.6e-3, HR = 0.93 [0.89–0.97]; TCGA-SKCM-M: *n* = 338, *p* = 8.6e-3, HR = 0.94 [0.90–0.98]) (Figure s2b).

High expression of *HAVCR2* was significantly correlated with improved overall survival both in primary and metastatic melanomas (TCGA-SKCM: *n* = 444, *p* = 9.7e-6; TCGA-SKCM-M: *n* = 347, *p* = 2.5e-4), reinforcing its role as a protective prognostic factor. DSS analysis similarly demonstrated that increased *HAVCR2* expression was associated with better prognosis in SKCM (TCGA-SKCM: *n* = 434, *p* = 0.01; TCGA-SKCM-M: *n* = 338, *p* = 0.02). Furthermore, PFI analysis supported this trend, with high HAVCR2 expression indicating favorable patient outcomes (TCGA-SKCM: *n* = 434, *p* = 0.01; TCGA-SKCM-M: *n* = 338, *p* = 0.02) (Figure s2c).

To evaluate whether the prognostic associations of *LAG3*, *TIGIT* and *HAVCR2* reflect immune-cell abundance, we performed multivariable Cox proportional hazards models adjusting for ImmuneScore and StromalScore derived from the ESTIMATE algorithm, along with available clinical covariates (age at diagnosis and AJCC pathologic stage). Our results show that both LAG3 and TIGIT showed borderline independent associations with improved overall survival after adjustment and FDR correction, indicating that their prognostic value is not fully explained by underlying immune infiltration. Specifically, higher *LAG3* expression was independently associated with reduced mortality risk (HR = 0.87; 95% CI, 0.77–0.98; FDR-adjusted *p* < 0.05), and *TIGIT* demonstrated a similar independent association (HR = 0.87; 95% CI, 0.76–0.99; FDR-adjusted *p* < 0.05). In contrast, *HAVCR2* did not retain statistical significance after FDR adjustment (HR = 0.89; 95% CI, 0.72–1.11; FDR-adjusted *p* > 0.05), suggesting that its apparent prognostic signal is more strongly linked to overall immune content within the tumor microenvironment.

To assess whether immune infiltration modifies these associations, we conducted stratified Cox analyses using median-split ImmuneScore categories. Within immune-high tumors, all three genes showed significant associations with improved overall survival even after FDR correction, indicating that their prognostic relevance is strongest in tumors characterized by substantial immune infiltration. However, none of the three genes remained significant within the immune-low subgroup following FDR adjustment, implying that the prognostic value of these markers is largely contingent upon a highly immunogenic tumor microenvironment. Collectively, these findings demonstrate that while *LAG3* and *TIGIT* exhibit modest but independent prognostic value in multivariable models, the prognostic associations of all three genes are substantially amplified in immune-rich skin cutaneous melanomas (Suppl. Tables 1–2 & Figure s3).

### Association between methylation and *LAG*3, *TIGIT* and *HAVCR2* expression

**Fig. 4:**
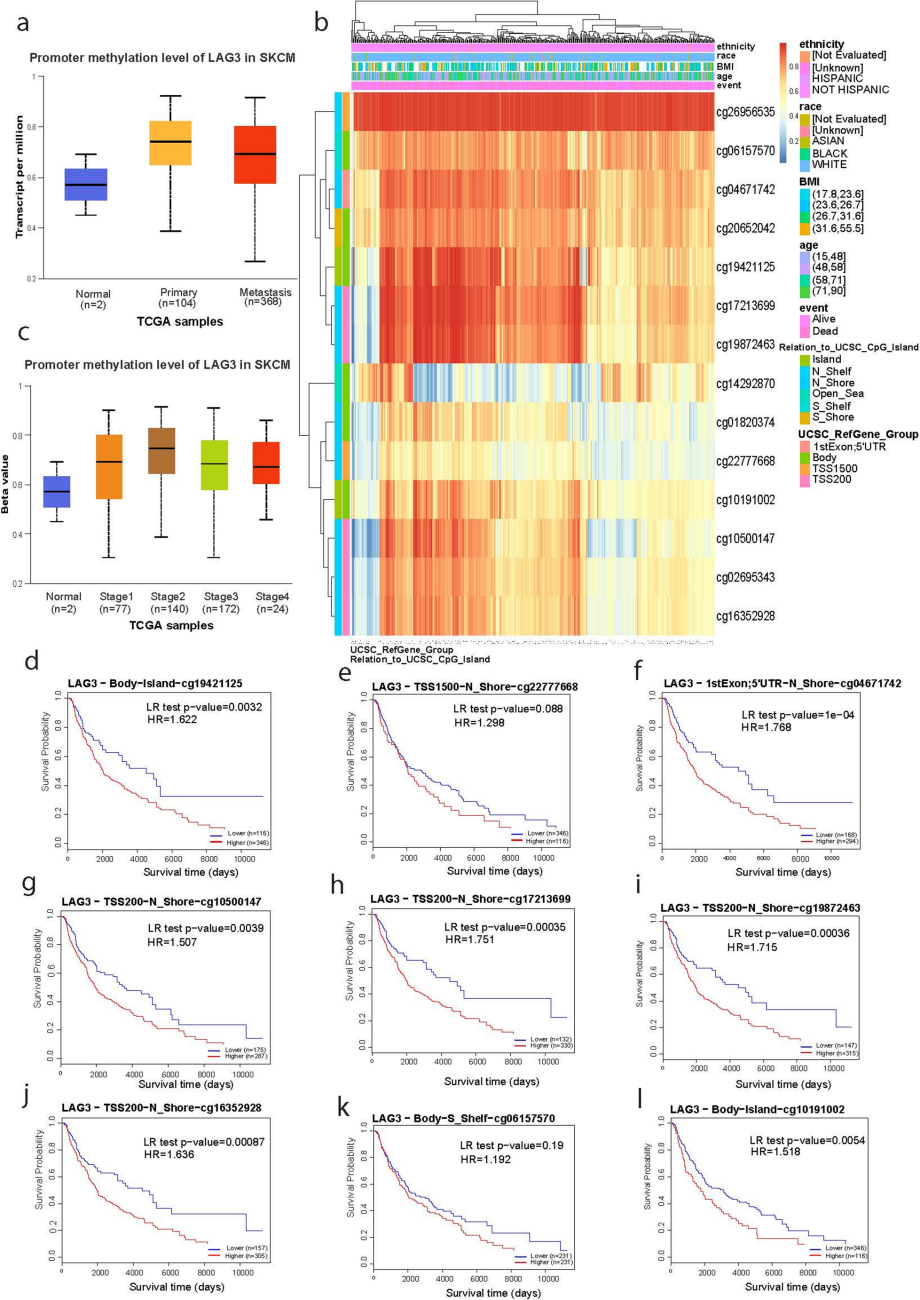
*LAG3* methylation and prognostic relevance in skin melanoma. (**a**) *LAG3* promoter methylation was significantly higher in primary (and metastatic) skin melanomas compared to the normal skin tissues (*p* < 0.0001). (**b**) CpG site-specific methylation analysis of *LAG3*. From high (red) to low (blue); ethnicity, race, age, event, relation_to_UCSC_CpG_island and UCSC_RefGene_Group are displayed with colored indications. (**c**) Stage 1–4 skin melanomas showed highest *LAG3* methylation levels compared to the normal skin, but no significant difference was noted among them. (**d**-**l**) The Kaplan-Meier curves show the survival and prognostic significance of methylation levels at 9 CpG sites in *LAG3* (cg10191002, cg26956535, cg06157570, cg04671742, cg20652042, cg19421125, cg17213699, cg19872463, cg14292870, cg01820374). Hypomethylation at most of these sites correlated with significantly better patient survival (*p* < 0.05). Unless otherwise noted, all p values are FDR-adjusted. Patients were stratified into high- and low-methylation groups based on the median methylation levels of each gene

Next, we aimed to investigate whether *LAG3*, *TIGIT* and *HAVCR2* are closely linked to DNA methylation at their promoter or gene body regions. Based on methylation data from TCGA-SKCM, we noted that the promoter methylation of *LAG3* was higher both in primary and metastatic melanomas compared to the normal skin tissues (*p* < 0.001) (Fig. 4a). We show that methylation (beta values) from at least seven methylation probes, cg26956535, cg06157570, cg04671742, cg20652042, cg19421125, cg17213699 and cg19872463, were negatively correlated with the expression level of *LAG3*, irrespective of the patients’ ethnicity, race, BMI or age. These high methylation levels were mainly related with “shores” and “shelves”, i.e., regions flanking CpG islands. Shores are usually within 2 kb of a CpG island, and shelves are between 2 and 4 kb away; as well as “open sea”, i.e., regions not being near CpG islands (typically >4 kb away) and having a very low density of CpG dinucleotides (Marzese and Hoon 2015). These regions were *N_shelf*,* N_shore*,* open_sea*,* S_shelf* (cg26956535, cg06157570, cg04671742), but also *Island* (cg19421125) and *S_shore* (cg20652042) CpG islands (Fig. 4b). We also found that promoter methylation of *LAG3* does not differ significantly across skin melanomas of stage 1–4 (Fig. 4c). Additionally, some methylation probes showed a correlation between *LAG3* hypomethylation and better patient survival (cg19421125, *p* = 0.0032; cg04671742, *p* = 1e-04; cg10500147, *p* = 0.0039; cg17213699, *p* = 0.00035; cg19872463, *p* = 0.00036; cg16352928, *p* = 0.00087; cg10191002, *p* = 0.0054) (Fig. 4d-l).

**Fig. 5:**
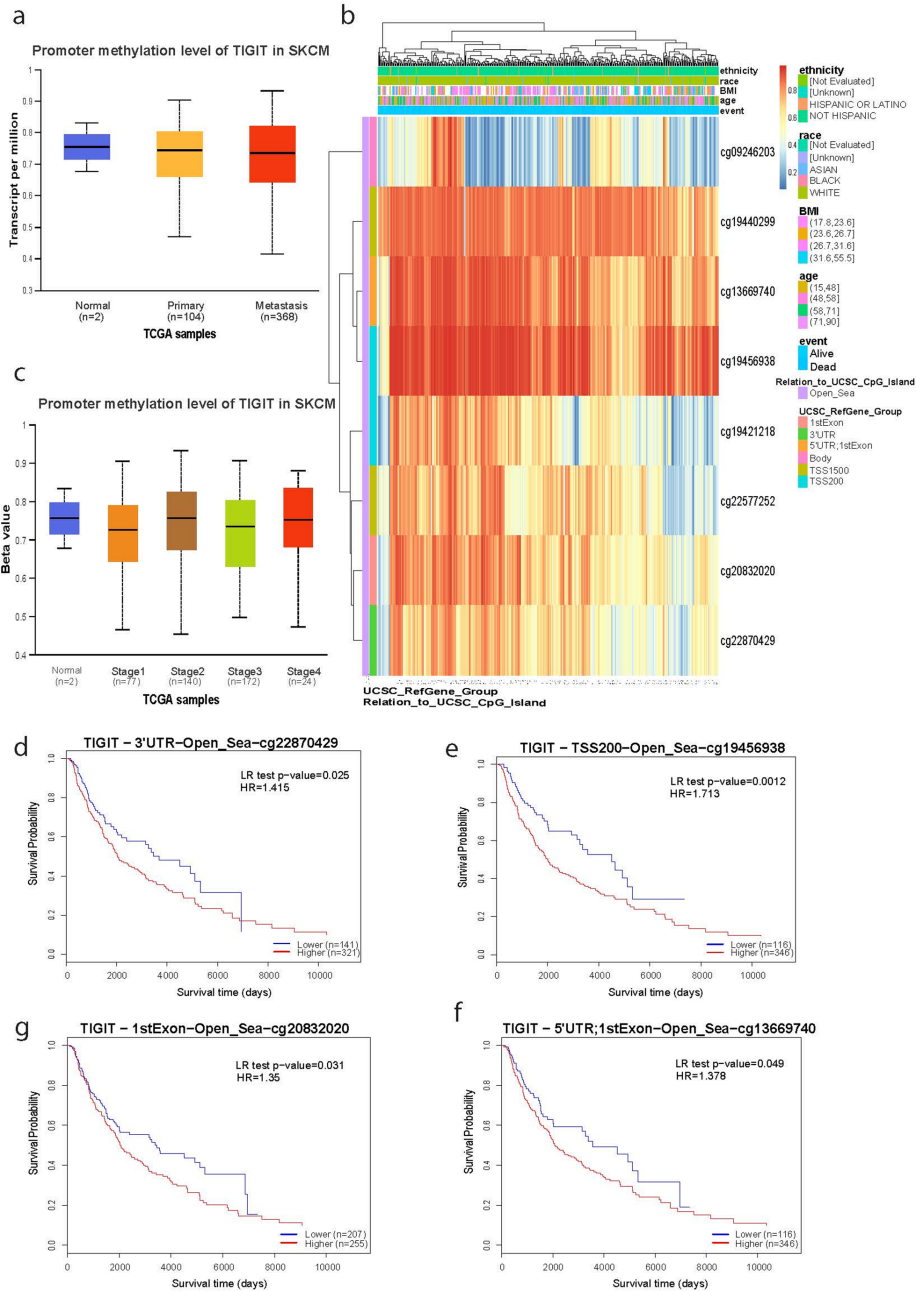
*TIGIT* methylation and prognostic prevalence in skin melanoma. (**a**) Promoter methylation of *TIGIT* did not different between primary (or metastatic) skin melanoma and the normal skin. (**b**) CpG site-specific methylation analysis of *TIGIT* (8 CpG sites: cg09246203, cg19440299, cg1366974, cg19456938, cg19421218, cg22577252, cg20832020, cg22870429). From high (red) to low (blue); ethnicity, race, age, event, relation_to_UCSC_CpG_island and UCSC_RefGene_Group are displayed with colored indications. (**c**) Promoter methylation of *TIGIT* did not differ across different stages of skin melanoma. (**d**) The Kaplan-Meier curves show that hypomethylation at 4 CpG sites in *TIGIT* (cg22870429, cg19456938, cg20832020, cg13669740) is correlated with better patient survival (*p* < 0.05). Unless otherwise noted, all p values are FDR-adjusted. Patients were stratified into high- and low-methylation groups based on the median methylation levels of each gene

*TIGIT* promoter methylation on the other hand, did not differ between primary (or metastatic) skin melanomas and the normal skin (Fig. 5a). We also show that methylation from at least three CpG island probes (cg19440299, cg13669740 and cg19456938) were negatively correlated with *TIGIT* expression. All these methylation markers were related with “open_sea” areas and their surrounding “shores” and “shelves” (Fig. 5b). In addition, *TIGIT* promoter methylation levels did not differ across tumor stages (Fig. 5c). Furthermore, some methylation probes showed a correlation between *TIGIT* hypomethylation and better patient survival in skin melanoma (cg22870429, *p* = 0.025; cg19456938, *p* = 0.0012; cg20832020, *p* = 0.031; cg13669740, *p* = 0.049) (Fig. 5d-f).

**Fig. 6:**
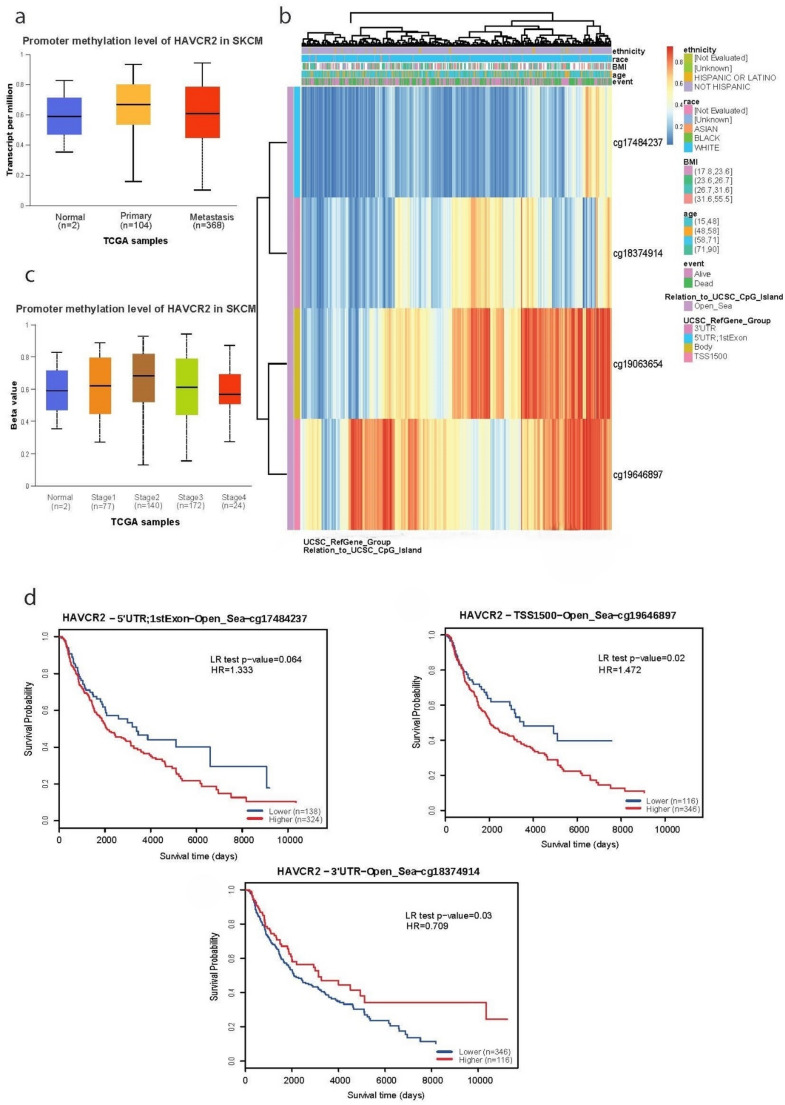
*HAVCR2* methylation and prognostic relevance in skin melanoma. (**a**) Promoter methylation levels of *HAVCR2* in normal, primary and metastatic skin melanoma. *HAVCR2* methylation levels were significantly elevated in SKCM tissues compared to normal controls. (**b**) CpG site-specific methylation analysis of *HAVCR2* (4 CpG sites). From high (red) to low (blue); ethnicity, race, age, event, relation_to_UCSC_CpG_island and UCSC_RefGene_Group are displayed with colored indications. (**c**) Stage-specific promoter methylation levels of *HAVCR2* did not differ significantly across skin melanomas of different tumor stages. (**d**) The Kaplan-Meier curves survival curves show that hypomethylation at cg19646897 and cg17484237 and hypermethylation at cg18374914 are associated with better survival (*p* < 0.05, *p* = 0.064 and *p* = 0.03, respectively). Unless otherwise noted, all p values are FDR-adjusted. Patients were stratified into high- and low-methylation groups based on the median methylation levels of each gene

Similar to *LAG3*, *HAVCR2* promoter methylation was higher in primary (and metastatic) melanomas compared to the normal skin (*p* < 0.05) (Fig. 6a). We also analyzed 4 CpG sites at “open sea” regions and found high methylation in two of them (cg19063654 and cg19646897) being negatively correlated with the gene’s expression (Fig. 6b). In terms of tumor staging, we found no significant differences in *HAVCR2* promoter methylation (Fig. 6c). Interestingly, survival time and probability varied across the methylation in the four CpG sites (cg22870429, cg19456938, cg20832020 and cg13669740). In specific, hypomethylation at cg19646897 was associated with significantly better patient survival (*p* = 0.02). Conversely, patients with hypomethylation at cg18374914 was correlated with worse patient survival (*p* = 0.03) (Fig. 6d).

### Prediction of MiRNAs targeting *LAG*−3, *TIGIT* and *HAVCR2*

Melanoma phenotype and progression are influenced by a multifactorial interaction of various regulatory molecules with microRNAs exhibiting a crucial role in these molecular networks(Singh et al. 2020). Therefore, we used several target gene prediction programs to identify miRNAs binding to *LAG3*, *TIGIT* and *HAVCR2*. By combining the data from miRWalk, miRDB, and miRabel databases, we identified a total of 10 miRNAs targeting *LAG3* (hsa-miR-2467-5p, hsa-miR-3064-5p, hsa-miR-3160-5p, hsa-miR-3614-5p, hsa-miR-4695-3p, hsa-miR-6726-5p, hsa-miR-6762-3p, hsa-miR-6842-3p, hsa-miR-8083 and hsa-miR-92a-1-5p) and 509 miRNAs targeting *TIGIT* (Suppl. Table 3), all being commonly detected across all three sources. On the other hand, no miRNAs were commonly detected across the three databases to bind *HAVCR2*.

### Relationship between *LAG*3, *TIGIT* and *HAVCR2* expression and immune infiltration

Tumor cells develop within a complex tumor microenvironment essential for their survival. This environment includes the tumor stroma, which contains various non-cancerous cells, including tumor-infiltrating immune cells (TIICs) being important for cancer progression (Azimi et al. 2012). The grade of tumor-infiltrating lymphocytes (TIL) is an independent predictor of survival and sentinel lymph node (SLN) status in melanoma patients (Azimi et al. 2012). Given this strong prognostic link, we investigated whether the expression of *LAG3*, *TIGIT* and *HAVCR2* is associated with the degree and composition of immune infiltration in skin melanoma.

**Fig. 7:**
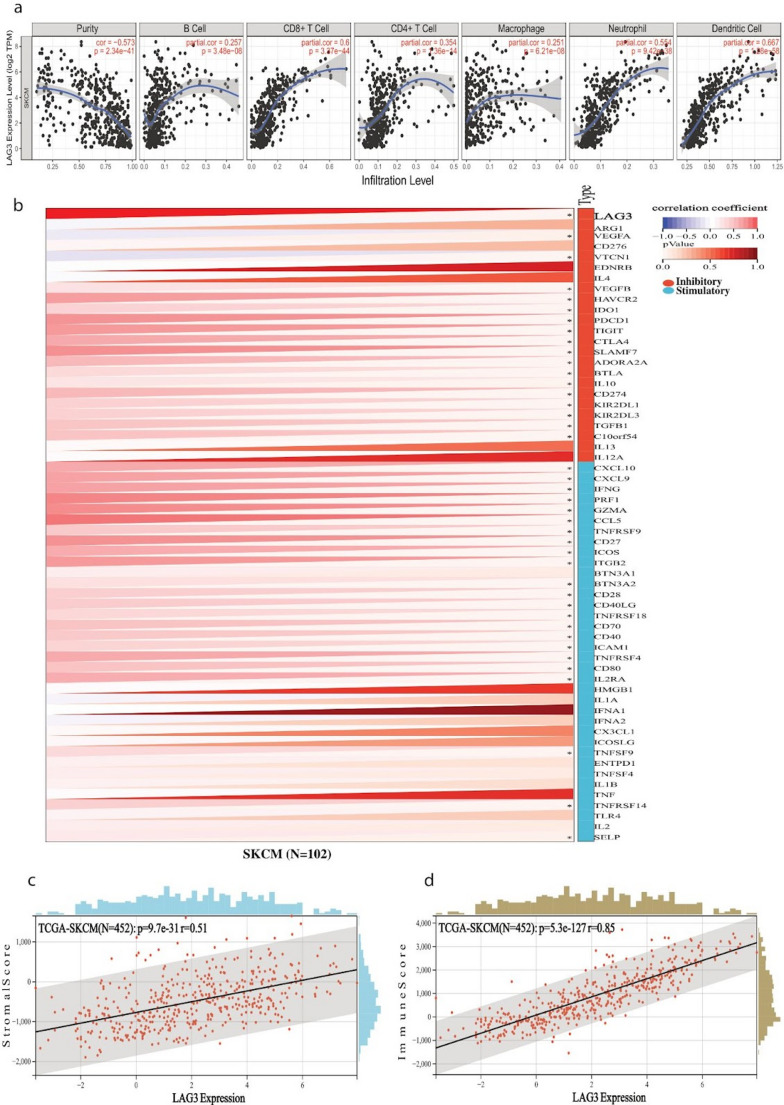
(**a**) Pearson’s correlation between *LAG3* expression and immune cell infiltration (B cells, CD8 + T cells, CD4 + T cells, macrophages, neutrophils, and dendritic cells) in skin melanoma. Tumor purity was adjusted for analysis. All displayed correlations are statistically significant (*p* < 0.001). (**b**) Pearson’s correlation between *LAG3* expression and immune checkpoint genes (inhibitory and stimulatory) expression across skin melanoma, using the TCGA TARGET GTEx dataset for SKCM. Expression values were log_2_-transformed and filtered to include only tumor samples. Significant associations highlight the immunomodulatory potential of *LAG3* for SKCM. (**c**-**d**) Pearson correlation between *LAG3* expression and immune (**c**) or stromal scores (**d**) in skin melanoma using the ESTIMATE algorithm. *LAG3* expression was positively correlated with both immune infiltration metrics, indicating higher *LAG3* expression is linked to increased immune and stromal cell infiltration in the TIME. Unless otherwise noted, all p values are FDR-adjusted

**Fig. 8:**
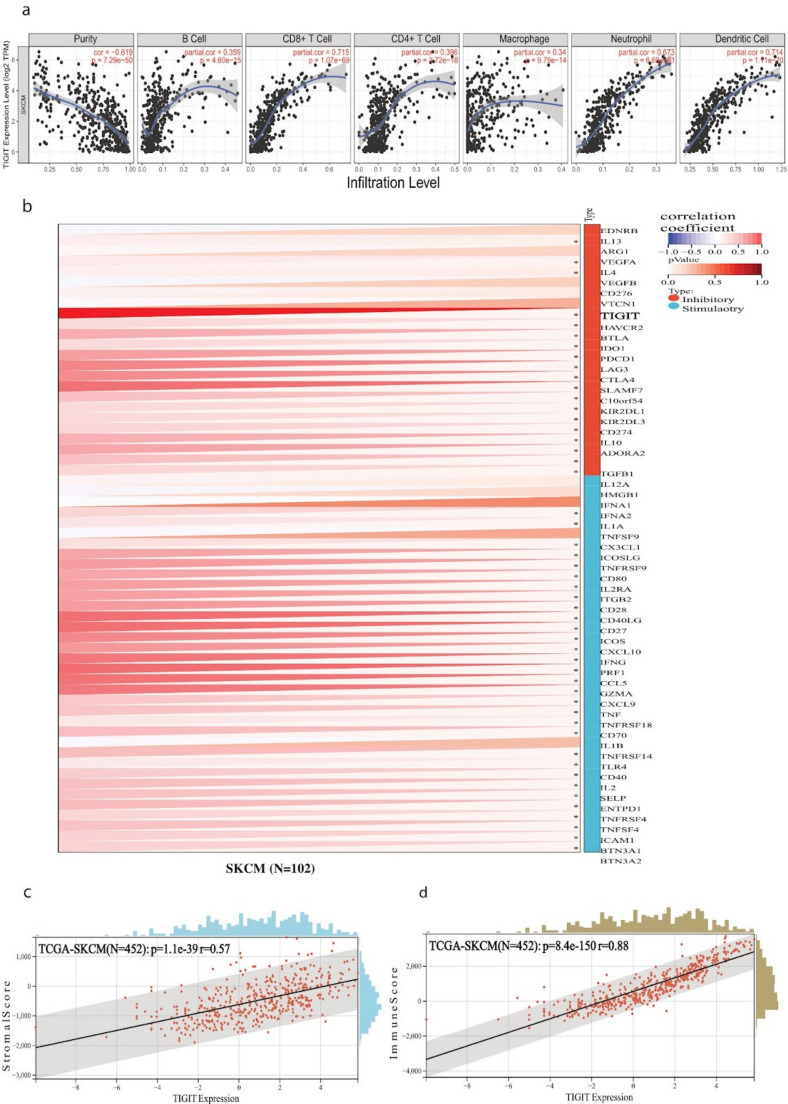
(**a**) Pearson’s correlation between *TIGIT* expression and immune infiltration in skin melanoma. Tumor purity was adjusted for analysis. All displayed correlations are statistically significant (*p* < 0.001). (**b**) Pearson correlation between *TIGIT* expression and immune checkpoint genes (inhibitory and stimulatory) expression in skin melanoma, using the TCGA TARGET GTEx dataset for SKCM. Expression values were log_2_-transformed and filtered to include only tumor samples. Significant associations highlight the immunomodulatory potential of *TIGIT* for SKCM. (**c**-**d**) Pearson correlation between *TIGIT* expression and immune (**c**) or stromal scores (**d**) in skin melanoma using the ESTIMATE algorithm. TIGIT expression was positively correlated with both scores, indicating higher *TIGIT* expression is linked to increased immune and stromal cell infiltration in the TIME. Unless otherwise noted, all p values are FDR-adjusted

**Fig. 9:**
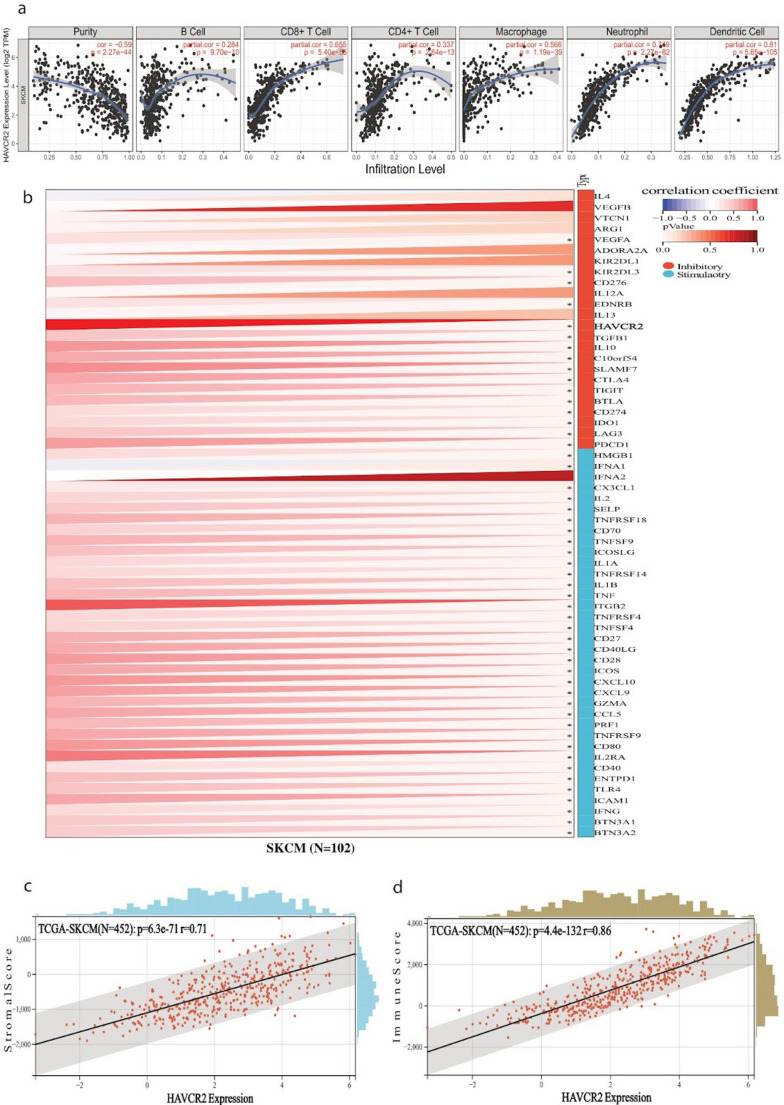
(**a**) Pearson’s correlation between *HAVCR2* expression and immune infiltration in skin melanoma. Tumor purity was adjusted for analysis. All displayed correlations are statistically significant (*p* < 0.001). (**b**) Pearson’s correlation between *HAVCR2* expression and immune checkpoint genes (inhibitory and stimulatory) expression in skin melanoma, using the TCGA TARGET GTEx dataset for SKCM. Expression values were log_2_-transformed and filtered to include only tumor samples. Significant associations highlight the immunomodulatory potential of *HAVCR2* for SKCM. (**c**-**d**) Pearson’s correlation between *HAVCR2* expression and immune (**c**) or stromal scores (**d**) in skin melanoma using the ESTIMATE algorithm. *HAVCR2* expression was positively correlated with both scores, indicating higher *HAVCR2* expression is linked to increased immune and stromal cell infiltration in the TIME. Unless otherwise noted, all p values are FDR-adjusted

To assess these relationships, we performed Pearson’s correlation analyses between gene expression levels and immune cell infiltration estimates, adjusting for tumor purity using TIMER 2.0 to reduce confounding. *LAG3* expression demonstrated a robust positive correlation with several immune cell populations, including CD8⁺ T cells, neutrophils, macrophages, monocytes, and dendritic cells. Importantly, all three genes—*LAG3*, *TIGIT*, and *HAVCR2*—were significantly and positively correlated with infiltration of B cells, CD8⁺ T cells, CD4⁺ T cells, macrophages, neutrophils, and dendritic cells (Figs. 7a, 8a and 9a), suggesting a broad immunological association. Interestingly, certain associations appeared to be context-dependent. *TIGIT* showed a marked correlation with CD4⁺ T cell and B cell infiltration only in metastatic melanomas, whereas *HAVCR2* exhibited a negative correlation with B cell infiltration in the metastatic setting. These findings point toward potential differences in immune checkpoint–immune cell relationships between primary and metastatic disease, which could have implications for understanding immune escape mechanisms in advanced melanoma.

We next sought to determine whether these genes are co-expressed with other immune checkpoint molecules. Using a large-scale pan-cancer transcriptomic dataset from UCSC, we examined correlations between *LAG3*, *TIGIT* and *HAVCR2* and 60 immune checkpoint genes. Pearson correlation analysis revealed widespread, statistically significant associations. For example, *LAG3* was strongly correlated with a range of inhibitory checkpoint markers, including *VEGFA/B*,* VTCN1*,* HAVCR2*,* IDO1*,* PDCD1*,* TIGIT*,* CTLA4*,* SLAMF7*,* ADORA2A*,* BTLA*,* IL10*,* CD274*,* KIR2DL1*,* KIR2DL3*,* TGFB1* and *C10orf54*. In addition, it was positively correlated with multiple stimulatory immune regulators such as *CXCL10*,* CXCL9*,* IFNG*,* PRF1*,* GZMA*,* CCL5*,* TNFRSF4*,* TNFRSF9*,* TNFRSF18*,* CD27*,* ICOS*,* ITGB2*,* BTN3A1/2*,* CD28*,* CD80*,* IL2RA* and *IFNA1*/*2* (Fig. 7b). Similar but distinct patterns of correlation were observed for *TIGIT* and *HAVCR2* (Figs. 8b and 9b), further highlighting their potential roles as central modulators within the melanoma immune landscape.

To further evaluate the broader immune context associated with *LAG3*, *TIGIT* and *HAVCR2* expression, we employed the ESTIMATE algorithm to calculate stromal and immune scores, which provide indirect measures of non-tumor cell infiltration within the tumor mass. All three genes displayed strong, statistically significant positive correlations with both stromal and immune scores (Figs. 7c–d, 8c–d and 9c–d). This indicates that higher expression of these immune checkpoints is consistently linked to increased immune cell presence and stromal enrichment in the tumor immune microenvironment (TIME).

Taken together, these findings suggest that *LAG3*, *TIGIT* and *HAVCR2* are not only markers of immune checkpoint activation but are also tightly associated with a highly immune-infiltrated melanoma microenvironment. The fact that these associations persist across multiple immune cell types and checkpoint molecules supports the notion that these genes participate in complex immunoregulatory networks in melanoma. Furthermore, the context-specific correlations observed in metastatic tumors point toward dynamic changes in immune regulation during disease progression, which may influence therapeutic responsiveness to immune checkpoint blockade.

## Discussion

In this study, we conducted an integrative, bioinformatics analysis utilizing various publicly available platforms to evaluate the expression profile, prognostic significance and immune infiltration patterns of *LAG3*, *TIGIT* and *HAVCR2* in skin melanoma. We also analyzed their methylation levels and potential miRNA-mediated post-transcriptional regulation to identify unexplored molecular biomarkers.

Ιn agreement with previous findings, we show that all three genes exhibit significantly higher expression in skin melanoma. In a pan-cancer analysis, Wen et al. (Wen et al. 2021) observed *TIGIT* upregulation in skin melanoma, reflecting an immunosuppressive tumor microenvironment being enriched in T-cell infiltrates and immune checkpoints. Notably, no major differences were observed between primary and metastatic melanoma for these immune checkpoints in some cohorts, indicating that high expression is a consistent feature across disease stages. Similarly, Georgoulias et al. previously reported markedly higher *LAG3*, *TIGIT* and *HAVCR2* mRNA levels in melanoma tissues (Georgoulias and Zaravinos 2022).

We also revealed that the expression of *LAG3*,* TIGIT* and *HAVCR2* is positively correlated with infiltration of most immune cells in skin melanoma. Specifically, *LAG3* expression was associated with infiltration of CD8 + T cells, neutrophils, macrophages, and dendritic cells, but not with CD4 + T cells or B cells. In addition, *TIGIT* was associated with infiltration of CD4 + T cells and B cells only in metastatic tumors, while *HAVCR2* was negatively associated with infiltration of B cells in metastatic melanomas. These findings align with previous reports identifying those immune checkpoints as critical regulators of T-cell exhaustion and immune escape in skin melanoma.

Consistent with our findings, Andrews et al. (2022), reported that elevated *LAG3* expression was associated with infiltration of exhausted CD8 + T cells, highlighting its contribution to immune resistance and supporting that its blockade may enhance T cell function and thus therapeutic responses in the disease (Andrews et al. 2022). Importantly, Lee et al. (2019), showed by immunohistochemistry that LAG3 and TIGIT protein levels strongly correlate with PD-1 in melanoma-infiltrated lymphocytes, consistently with co-expression on exhausted CD8 T cells (Lee et al. 2019). Similarly, single-cell RNA-sequencing of melanoma TILs confirms that *HAVCR2* is highly expressed in exhausted CD8 + T cells. In addition, Naimy et al., found that *LAG3* and *TIGIT* are highly expressed in melanomas with brisk TILs compared to TIL-negative tumors (Naimy et al. 2024). Another recent study highlighted that tumor-infiltrating γδ cells in melanoma lesions express LAG3, HAVCR2 and PD-1 (Simone et al. 2024). Additionally, Gide et al. reported that the LAG3 protein is localized to TILs and dendritic cells in 81% of metastatic melanoma samples (Gide et al. 2023), providing a strong indication that dendritic cells in the tumor can also express *LAG3*, potentially contributing to local immunoregulation.

Furthermore, we found a positive correlation between high *LAG3*, *TIGIT* and *HAVCR2* expression and improved patient survival both in primary and metastatic tumors. This is consistent with a study that reported that patients with high expression of these genes in the tumor had significantly better overall and disease-free survival. A pan-cancer investigation focusing on *HAVCR2*, similarly found that the gene acts as a protective prognostic factor in skin melanoma – higher *HAVCR2* expression was associated with longer overall, disease-specific, and progression-free survival (Gide et al. 2018). Another recent study by Gide et al. (Gide et al. 2023) evaluated immune checkpoints in terms of PFS, and reported that baseline immune checkpoint expression could predict clinical outcomes. Specifically, among metastatic melanoma patients treated with a combination therapy of anti-LAG3 and anti-PD-1 monoclonal antibodies, those with ≥ 1% LAG3 cells in the tumor demonstrated significantly lower PFS compared to those with low LAG3 expression (Gide et al. 2023). In addition, patients who responded to the treatment showed higher LAG3 in TIL and dendritic cell infiltration at baseline, suggesting a more immune-infiltrated tumor microenvironment primed for response. These findings suggest that LAG3 along with other co-inhibitory receptors, may serve as valuable biomarkers for identifying tumors more likely to be responsive to combination immunotherapies, potentially demonstrating a longer PFS under treatment (Gide et al. 2023).

Although high expression of *LAG3*, *TIGIT* and *HAVCR2* is associated with improved prognosis, this likely reflects an inflamed tumor microenvironment enriched in activated T cells. Their inhibitory function, however, contributes to exhaustion of these same T cells. Thus, while high expression marks favorable pre-existing immunity, therapeutic blockade is expected to further potentiate anti-tumor responses by relieving inhibitory signaling on activated lymphocytes.

Some studies also noted contrasting findings. Specifically, a comprehensive analysis observed that patients with low *LAG3*,* HAVCR2* and *PD-1* expression had better 1-year OS in melanoma (Huuhtanen et al. 2023). Similarly, a study by Kim et al. (2020), reported that in cutaneous melanoma the concurrent presence of high PD-1 and LAG3 expression was associated with increased M2-type tumor-associated macrophages. When both high M2-TAMs and immune checkpoint molecules were present, they acted as poor prognostic factors for cutaneous melanoma (Kim et al. 2020). These results indicate that the prognostic role of *LAG3*,* TIGIT* and *HAVCR2* is context-dependent. While high expression may show an immune-infiltrative tumor microenvironment, which is often associated with better outcomes, it also indicates T cell exhaustion, which could facilitate immune evasion and tumor progression.

Due to the fact that *LAG3*, *TIGIT* and *HAVCR2* are predominantly expressed by tumor-infiltrating immune cells, their bulk RNA expression largely reflects the extent of immune infiltration in melanoma. Our multivariable Cox models confirmed that immune infiltrates account for a substantial part of their prognostic signal, yet each gene retained a modest but measurable association with overall survival after adjusting for these measures of the tumor microenvironment. Thus, their interpretation as prognostic biomarkers should occur within the broader immunologic context of the tumor microenvironment, rather than as isolated tumor-cell–intrinsic markers.

Epigenetic effects have a crucial role in the regulation of gene expression. Unlike the distribution of DNA methylation in normal cells, cancer cells present increased DNA methylation in CpG-rich regions and decreased DNA methylation in CpG-poor regions. Traditionally, gene promoter CpG islands hypermethylation has been largely studied in cancer, however, new studies show that aberrant methylation in regions other than gene promoter CpG islands may potentially be novel theranostic markers (Yang et al. 2014). In addition to gene-specific aberrant methylation, the characterization of tumor type- and stage-specific genome-wide DNA methylation patterns offers an opportunity to identify unexplored molecular biomarkers. Prior investigations have suggested that DNA methylation patterns and pigmentation gene expression could potentially mediate the relationship between genetic variants, pigmentation phenotypes, and the risk of skin cancer (Bonilla et al. 2021).

We further explored the potential regulatory mechanisms regulating *LAG3*, *TIGIT* and *HAVCR2* expression in the disease, by analyzing their promoter methylation levels. *LAG3* exhibited higher promoter methylation in primary and metastatic melanomas, with variations across different stages of the disease. Interestingly, we showed that hypomethylation at several *LAG3* CpG sites was associated with an improved patient survival. These findings are consistent with the study of Fröhlich et al. (2020), who found that promoter methylation is inversely associated with *LAG3* mRNA expression in melanoma tumors. This study concluded that *LAG3* promoter hypomethylation leads to higher gene expression and is associated with greater immune cell infiltration and improved survival outcomes(Fröhlich et al. 2020).

Similarly, *TIGIT* methylation analysis revealed multiple differentially methylated sites influencing the gene’s expression and patient prognosis. A study demonstrated that DNA methylation regulates *TIGIT* expression in the skin melanoma microenvironment, which aligns with our results (Niebel et al. 2022). Notably, this study revealed that melanoma patients with low *TIGIT* methylation demonstrated longer overall survival, while low promoter methylation predicted improved progression-free survival under anti-PD-1 treatment (Niebel et al. 2022). This highlights the association between TIGIT methylation and prognosis in the disease, aligning with our findings.

For *HAVCR2* (*TIM-*3), the promoter methylation was also higher in primary (and metastatic) melanoma. We highlight four sites in the *HAVCR2* promoter with differential methylation, two with lower methylation, which was associated with longer survival and one with higher methylation, linked with better prognosis. These findings are consistent with the findings of Holderried et al. (2019), who reported that *HAVCR2* promoter methylation is inversely correlated with the gene’s expression in melanoma (Holderried et al. 2019). Melanoma patients with low *HAVCR2* promoter methylation showed significantly better overall survival. A comprehensive analysis across multiple cancers including skin melanoma, reinforcesthis correlation in *HAVCR2* (Li et al. 2022). Notably, *HAVCR2* is diferentially methylated across multiple tumor types and a significant association between this gene’s methylation levels and its expression has been observed. In addition, in immune-rich environments, deferential *HAVCR2* methylation has been linked with T-cell activation, indicating that demethylation can accompany *HAVCR2* upregulation on infiltrating T cells (Li et al. 2022). Overall, these findings demonstrate that the epigenetic regulation of these genes’ expression can shape the tumor immune microenvironment and influence patient prognosis.

Importantly, we explored gene-specific methylation in -non-CpG-rich island- genomic regions with a lower CpG density, since these have been shown to be functionally important for gene expression regulation. Although CpG shores and shelves exhibit a lower CpG density, they have proven to be functionally significant in the regulation of gene expression. For instance, CpG shores have been linked to cancer-, tissue-, and reprogramming-specific differentially methylated regions (C-DMRs, T-DMRs, and R-DMRs), with mechanistic implications for the expression of nearby genes (Irizarry et al. 2009; Doi et al. 2009). During cell differentiation, CpG shore methylation displays greater dynamism than CpG island methylation, underscoring its pivotal role in determining cell fate (Doi et al. 2009). Moreover, differential methylation patterns in CpG shelves and open sea regions have been associated with hepatocellular carcinoma (Shen et al. 2013) and metastatic melanoma (Marzese et al. 2014).

To further explore potential mechanisms of post-transcriptional regulation, we examined miRNAs that are predicted to target *LAG3*,* TIGIT* and *HAVCR2*. We paid attention to 10 miRNAs that were found to regulate *LAG3*, and 509 miRNAs for *TIGIT*, indicating different post-transcriptional control mechanisms. The no common miRNAs were identified across databases expression might reflect that this gene’s transcriptional control is mainly regulated by methylation. To date only a few miRNAs—such as miR-7704, miR-21-5p, and miR-16—have been associated with *LAG3* expression. Zhao et al. found that miR-7704 and miR-21-5p can regulate transcriptionally *LAG*3 (Zhao et al. 2022). In contrast, the extensive network of miRNAs that were predicted to regulate *TIGIT*, aligns with studies indicating that long non-coding RNAs (IncRNAs) such as MEG3 can modulate *TIGIT* expression by sponging miR-23a, thereby influencing CD4 + T cell activation (Wang et al. 2019).

Furthermore, the absence of significant miRNA interactions with *HAVCR2* in our analysis is notable, hinting towards other mechanisms regulating *HAVCR2* expression, such as methylation. Notably, one study reported that circPVT1 enhances *HAVCR2* expression by sponging miR-490-5p, thus promoting migration and invasion in osteosarcoma cells. This finding implies that *HAVCR2* may be regulated indirectly through competing endogenous RNA (ceRNA) networks rather than by direct miRNA binding (Zhou et al. 2024).

Finally, while our study provides valuable insights, there are some limitations to consider. First, our findings are based on publicly available datasets, thus experimental validation in patient-derived samples or preclinical models is needed. Additionally, while our study establishes correlations between gene expression, survival outcomes, and immune infiltration, further mechanistic studies are required to elucidate the precise functional roles of *LAG3*,* TIGIT* and *HAVCR2* in skin melanoma progression and immune modulation. Future research should aim on validating these findings in larger and independent cohorts of melanoma patients, through longitudinal studies monitoring treatment outcomes over time. Notably, multi-omics approaches including proteomics and RNA sequencing, will be beneficial to evaluate the therapeutic potential of *LAG3*,* TIGIT* and *HAVCR2* in skin melanoma.

## Conclusion

This study demonstrates that *LAG3*,* TIGIT* and *HAVCR2* are associated with immune cell infiltration and immune regulatory pathways in skin melanoma. While Relatlimab is an already clinically validated anti-LAG3 mab, the consistent associations observed for *TIGIT* and *HAVCR2* suggest that these genes may serve as promising targets for immunotherapy. Future studies should focus on investigating the roles of *TIGIT* and *HAVCR2* in tumor-immune interactions and assessing their potential in combination with existing immune checkpoint inhibitors to improve therapeutic outcomes in skin melanoma.

## Supplementary Information

Below is the link to the electronic supplementary material.

Supplementary Material 1 (DOCX 2.22 MB)

## Data Availability

TIMER (https://cistrome.shinyapps.io/timer/); CCLE (https://portals.broadinstitute.org/ccle); HPA (http://www.proteinatlas.org); CIBERSORT (http://cibersort.stanford.edu/); GEPIA (http://gepia.cancer-pku.cn/); Sangerbox (http://vip.sangerbox.com/home.html); UALCAN (http://ualcan.path.uab.edu); MethSurv (https://biit.cs.ut.ee/methsurv/); miRWalk (http://mirwalk.umm.uni-heidelberg.de/); miRDB (http://mirdb.org/); miRabel (https://tools4mirs.org/software/target_prediction/mirabel//); CancerMIRNome (https://ngdc.cncb.ac.cn/databasecommons/database/id/8011); Kaplan-Meier plotter (http://kmplot.com); STRING (http://string-db.org); GeneMANIA (http://www.genemania.org); cBioPortal (http://cbioportal.org); the UCSC Xena Browser (https://xena.ucsc.edu/); (CMap) (https://clue.io/).

## Declarations

### Generative AI and AI-assisted technologies in the writing process

During the preparation of this work the authors used ChatGPT to improve the readability and language of the manuscript. After using this tool/service, the authors reviewed and edited the content as needed and take full responsibility for the content of the published article.
