# Supplementary material for: Predicting skin melanoma progression via LAG-3, TIGIT and HAVCR2
Source: Funct Integr Genomics. 2026 Feb 26;26(1):53. doi: 10.1007/s10142-026-01832-0 (PMC12945989; doi:10.1007/s10142-026-01832-0)
Supplement: Supplementary file 1 — Supplementary Material 1 (DOCX 2.22 MB) [file 10142_2026_1832_MOESM1_ESM.docx]

**Supplementary files**

**Supplementary Figures:**


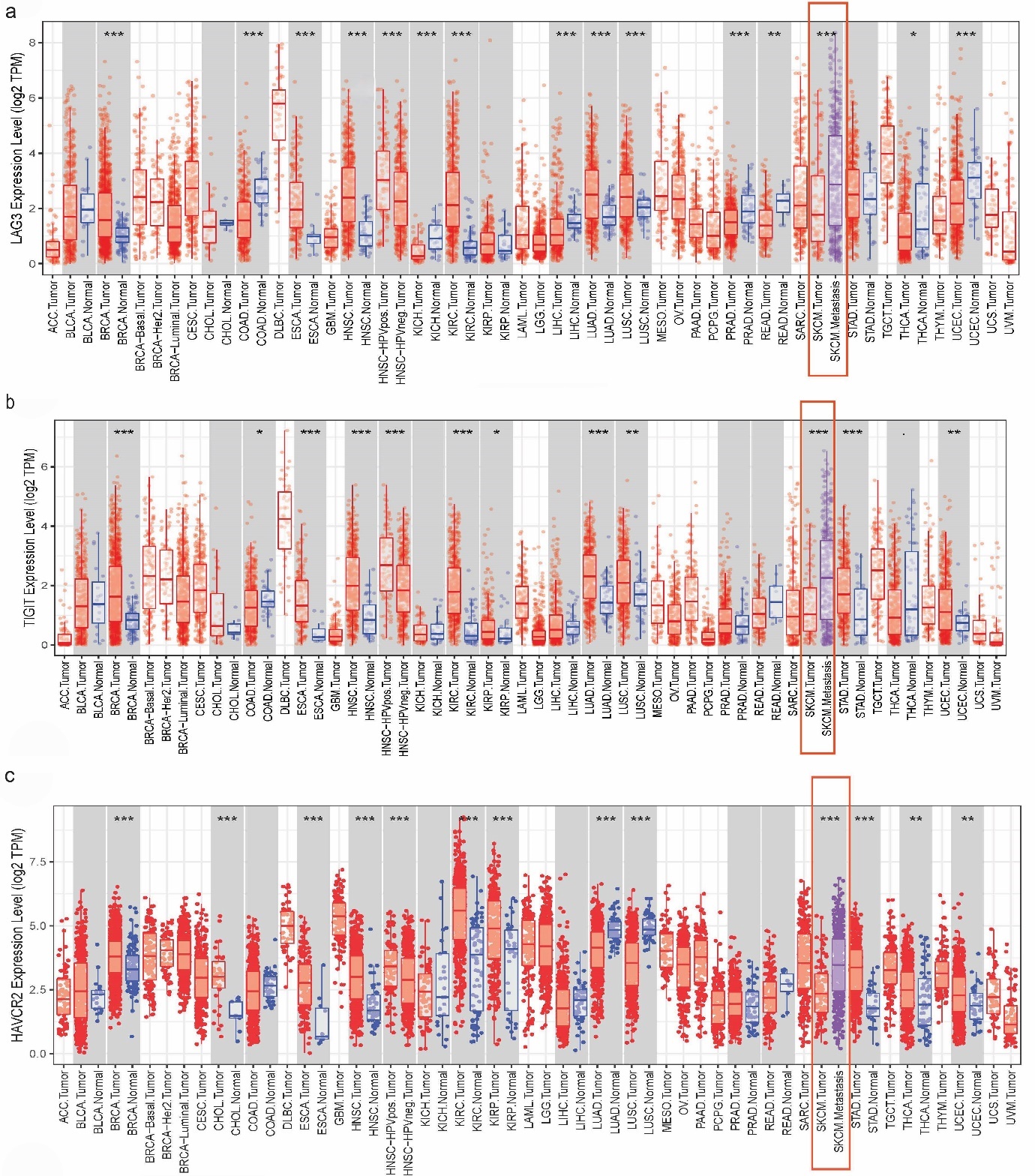


**Figure s1.** TIMER 2.0 confirmed the elevated expression of *LAG3* (a), *TIGIT* (b) and *HAVCR2* (c) in metastatic skin melanoma compared to primary melanoma (***, p<0.001; Wilcoxon rank-sum test).


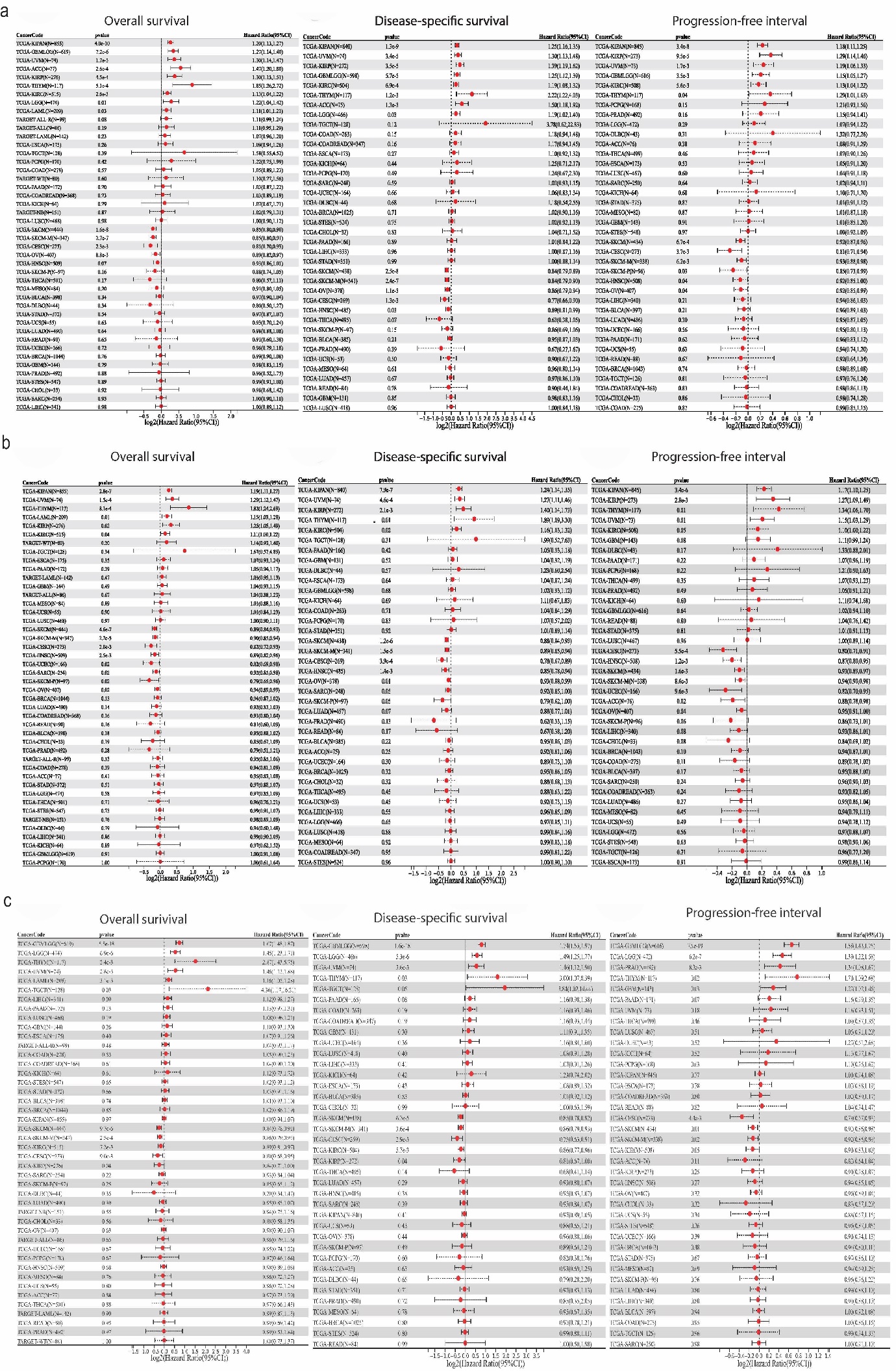
**Figure s2.** Overall, disease-specific, and progression-free interval survival analysis of *LAG3*, *TIGIT* and *HAVCR2* expression in pan-cancer, including skin melanoma (TCGA-SKCM).


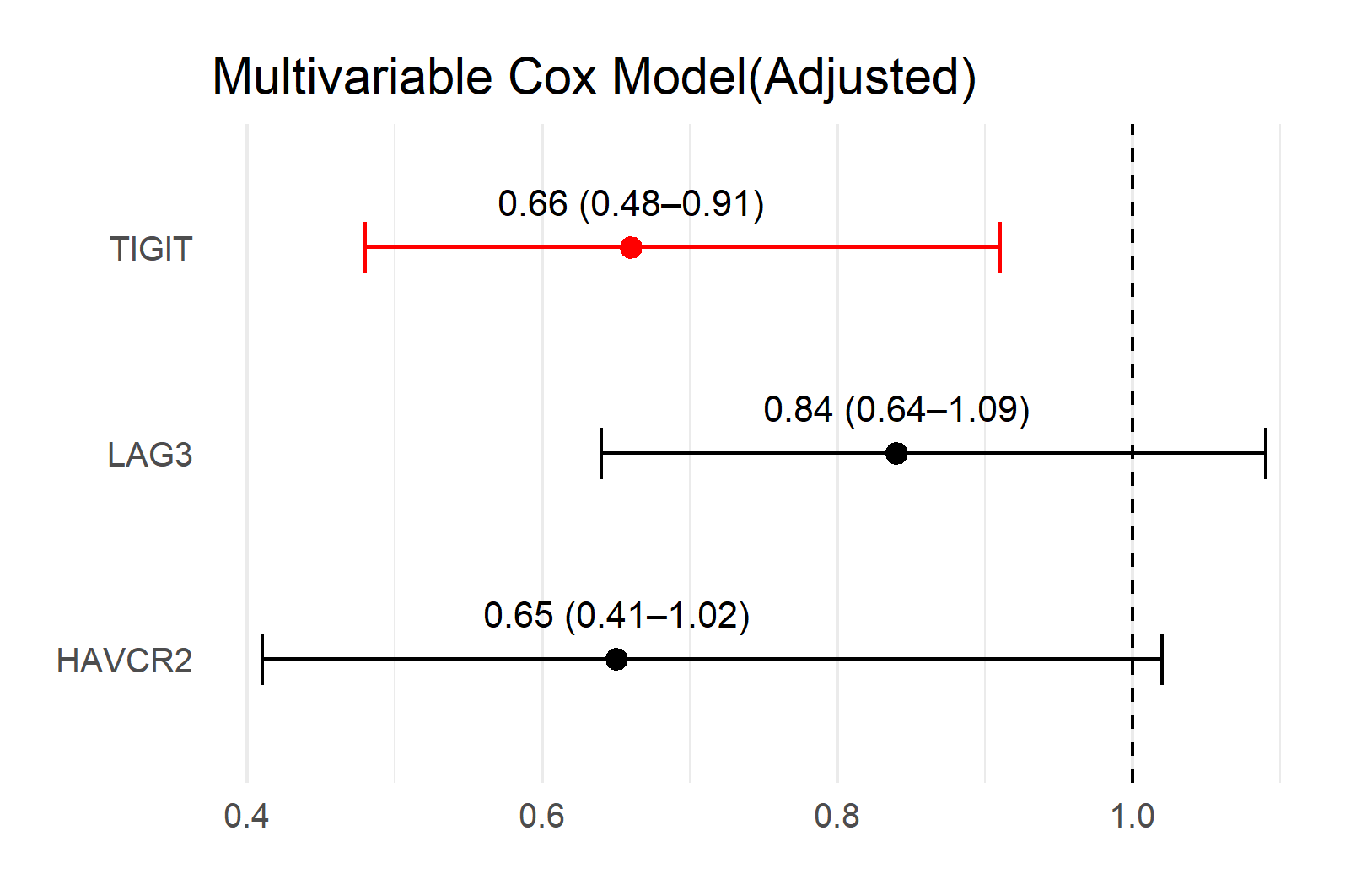

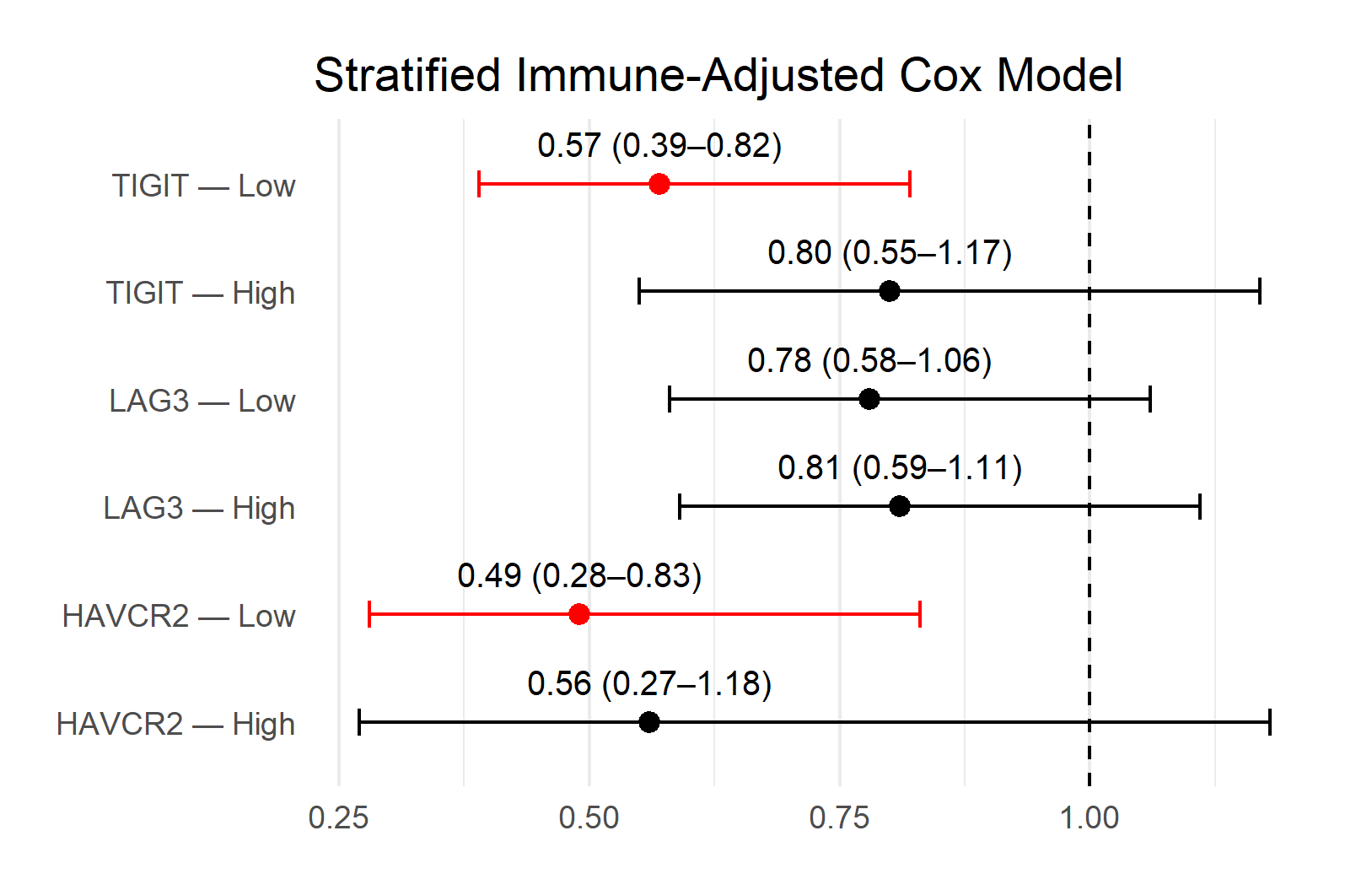


**Figure s3.** (left) Multivariable Cox model (adjusted) for TIGIT, LAG2 and HAVCR2. (right) Stratified immune-adjusted Cox model for (TIGIT, LAG3, HAVCR2) immune-high and immune-low tumors.

**Supplementary Tables:**

**Suppl. Table 1.** Multivariable Cox Regression.

| Gene | Hazard Ratio (95% CI) | p | significance row |
| --- | --- | --- | --- |
| **LAG3** | **0.87 (0.77–0.98)** | **0.026** | **true** |
| **TIGIT** | **0.87 (0.76–0.99)** | **0.035** | **true** |
| HAVCR2 | 0.89 (0.72–1.11) | 0.303 | false |
| Abbreviations: HR, hazard ratio; CI, confidence interval. | | | |
| P values are from multivariable Cox proportional hazards models adjusted for age, AJCC stage, immune score, and stromal score. | | | |

**Suppl. Table 2.** Immune-Stratified Cox Regression.

| Gene | ImmuneGroup | Hazard Ratio (95% CI) | p | significance row |
| --- | --- | --- | --- | --- |
| **LAG3** | **High** | **HR = 0.80 (95% CI 0.70–0.90)** | **<0.001** | **true** |
| LAG3 | Low | HR = 0.90 (95% CI 0.80–1.01) | 0.076 | false |
| **TIGIT** | **High** | **HR = 0.73 (95% CI 0.63–0.84)** | **<0.001** | **true** |
| TIGIT | Low | HR = 0.92 (95% CI 0.83–1.01) | 0.095 | false |
| **HAVCR2** | **High** | **HR = 0.71 (95% CI 0.56–0.89)** | **0.004** | **true** |
| HAVCR2 | Low | HR = 0.95 (95% CI 0.82–1.10) | 0.506 | false |
| Abbreviations: HR, hazard ratio; CI, confidence interval. | | | | |
| Stratified Cox models were performed separately within immune-high and immune-low groups. | | | | |
| P values < 0.05 were considered statistically significant. | | | | |

**Suppl. Table 3.** Target gene prediction using miRWalk, miRDB, and miRabel to identify miRNAs binding to LAG3, TIGIT and HAVCR2.

| mirnaid | refseqid | start | end | bindingp | accessibility | phylopstem | phylopflank | binding_region_length | position |
| --- | --- | --- | --- | --- | --- | --- | --- | --- | --- |
| hsa-miR-4800-5p | NM_173799 | 910 | 940 | 0.95385 | 0.10954 | 2.31313 | 1.78004 | 19 | 3UTR |
| hsa-miR-604 | NM_173799 | 1220 | 1237 | 0.96154 | 0.00108 | 0.34788 | 0.78278 | 17 | 3UTR |
| hsa-miR-1207-5p | NM_173799 | 1638 | 1660 | 0.96154 | 2.81E-06 | 0.04022 | 0.77789 | 22 | 3UTR |
| hsa-miR-1976 | NM_173799 | 1426 | 1471 | 0.96154 | 0.00351 | 0.99814 | 0.50257 | 22 | 3UTR |
| hsa-miR-3615 | NM_173799 | 1820 | 1842 | 0.96154 | 0.01144 | 0.06066 | 0.31069 | 22 | 3UTR |
| hsa-miR-3911 | NM_173799 | 1665 | 1683 | 0.96154 | 0.00692 | 0.91657 | 0.11713 | 18 | 3UTR |
| hsa-miR-3938 | NM_173799 | 1423 | 1443 | 0.96154 | 0.00093 | 1.26462 | 0.30312 | 20 | 3UTR |
| hsa-miR-5088-3p | NM_173799 | 1828 | 1852 | 0.96154 | 0.00158 | 0.30645 | 0.29741 | 24 | 3UTR |
| hsa-miR-6756-3p | NM_173799 | 1831 | 1852 | 0.96154 | 0.00158 | 0.44264 | 0.25091 | 21 | 3UTR |
| hsa-miR-6762-5p | NM_173799 | 1396 | 1428 | 0.96154 | 4.95E-05 | -0.33969 | 0.37654 | 32 | 3UTR |
| hsa-miR-7155-5p | NM_173799 | 1418 | 1436 | 0.96154 | 6.12E-06 | -0.0119 | 0.41692 | 18 | 3UTR |
| hsa-miR-4433b-3p | NM_173799 | 2713 | 2736 | 0.96154 | 0.00119 | 0 | 0 | 23 | 3UTR |
| hsa-miR-6814-5p | NM_173799 | 1704 | 1722 | 0.96923 | 0.00128 | 0.69903 | 0.10367 | 18 | 3UTR |
| hsa-miR-6894-5p | NM_173799 | 2263 | 2288 | 0.96923 | 0.00055 | 0 | 0 | 25 | 3UTR |
| hsa-miR-371b-3p | NM_173799 | 2139 | 2158 | 0.97436 | 0.00366 | 2.23877 | 1.13596 | 19 | 3UTR |
| hsa-miR-6858-3p | NM_173799 | 1818 | 1839 | 0.97436 | 0.066 | 0.24221 | 0.27 | 21 | 3UTR |
| hsa-miR-770-5p | NM_173799 | 1902 | 1924 | 0.97436 | 3.25E-05 | 1.01797 | 0.38574 | 22 | 3UTR |
| hsa-miR-499b-5p | NM_173799 | 959 | 976 | 0.97436 | 6.84E-06 | 1.34829 | 1.95134 | 17 | 3UTR |
| hsa-miR-10392-3p | NM_173799 | 1819 | 1839 | 0.98077 | 0.066 | 0.2395 | 0.26756 | 20 | 3UTR |
| hsa-let-7a-2-3p | NM_173799 | 1828 | 1848 | 1 | 0.00124 | 0.06066 | 0.31069 | 20 | 3UTR |
| hsa-let-7c-3p | NM_173799 | 1828 | 1848 | 1 | 0.00124 | 0.06066 | 0.31069 | 20 | 3UTR |
| hsa-let-7e-3p | NM_173799 | 1828 | 1848 | 1 | 0.00124 | 0.06066 | 0.31069 | 20 | 3UTR |
| hsa-miR-15a-5p | NM_173799 | 2744 | 2785 | 1 | 0.01263 | 0 | 0 | 22 | 3UTR |
| hsa-miR-15a-3p | NM_173799 | 1385 | 1427 | 1 | 4.05E-05 | 0.3435 | 0.23827 | 42 | 3UTR |
| hsa-miR-18a-3p | NM_173799 | 1454 | 1483 | 1 | 0.00047 | 0.32107 | 0.60414 | 29 | 3UTR |
| hsa-miR-22-3p | NM_173799 | 1031 | 1052 | 1 | 0.00141 | 0.85131 | 1.31995 | 21 | 3UTR |
| hsa-miR-28-3p | NM_173799 | 1464 | 1481 | 1 | 0.00027 | 0.07907 | 0.55152 | 17 | 3UTR |
| hsa-miR-29a-5p | NM_173799 | 1498 | 1547 | 1 | 0.00013 | 0.23727 | 0.74118 | 21 | 3UTR |
| hsa-miR-30a-3p | NM_173799 | 2437 | 2485 | 1 | 0.00562 | 0 | 0 | 21 | 3UTR |
| hsa-miR-31-5p | NM_173799 | 1092 | 1105 | 1 | 0.00145 | 0.96551 | 1.41784 | 13 | 3UTR |
| hsa-miR-31-3p | NM_173799 | 1356 | 1403 | 1 | 0.00115 | 0.29409 | 0.3227 | 21 | 3UTR |
| hsa-miR-93-5p | NM_173799 | 1082 | 1105 | 1 | 0.00145 | 0.57722 | 1.4778 | 23 | 3UTR |
| hsa-miR-93-3p | NM_173799 | 2428 | 2470 | 1 | 0.00181 | 0 | 0 | 21 | 3UTR |
| hsa-miR-96-3p | NM_173799 | 2078 | 2111 | 1 | 0.00359 | 0.74414 | 1.11676 | 22 | 3UTR |
| hsa-miR-99a-5p | NM_173799 | 1994 | 2041 | 1 | 0.04083 | 0.19351 | 0.27239 | 22 | 3UTR |
| hsa-miR-100-5p | NM_173799 | 1994 | 2041 | 1 | 0.04083 | 0.21728 | 0.26511 | 22 | 3UTR |
| hsa-miR-103a-2-5p | NM_173799 | 1445 | 1466 | 1 | 0.00031 | -0.07321 | 0.587 | 21 | 3UTR |
| hsa-miR-103a-1-5p | NM_173799 | 1445 | 1466 | 1 | 0.00031 | -0.07321 | 0.587 | 21 | 3UTR |
| hsa-miR-103a-1-5p | NM_173799 | 2426 | 2457 | 1 | 0.06753 | 0 | 0 | 31 | 3UTR |
| hsa-miR-105-3p | NM_173799 | 1677 | 1715 | 1 | 0.00023 | 0.51266 | 0.03515 | 38 | 3UTR |
| hsa-miR-30d-3p | NM_173799 | 2456 | 2485 | 1 | 0.00562 | 0 | 0 | 23 | 3UTR |
| hsa-miR-139-5p | NM_173799 | 2691 | 2737 | 1 | 0.00016 | 0 | 0 | 46 | 3UTR |
| hsa-miR-147a | NM_173799 | 773 | 799 | 1 | 0.00446 | 1.5462 | 1.97217 | 26 | 3UTR |
| hsa-miR-204-5p | NM_173799 | 1395 | 1442 | 1 | 0.00126 | -0.22548 | 0.50869 | 23 | 3UTR |
| hsa-miR-211-5p | NM_173799 | 1818 | 1852 | 1 | 0.00158 | 0.19871 | 0.3485 | 22 | 3UTR |
| hsa-miR-212-5p | NM_173799 | 1375 | 1392 | 1 | 0.00755 | 0.26397 | 0.15507 | 17 | 3UTR |
| hsa-miR-214-3p | NM_173799 | 851 | 875 | 1 | 0.16796 | 0.96318 | 1.91513 | 24 | 3UTR |
| hsa-miR-224-5p | NM_173799 | 2027 | 2047 | 1 | 0.13743 | 0.32127 | 0.30671 | 20 | 3UTR |
| hsa-miR-200b-5p | NM_173799 | 1305 | 1340 | 1 | 0.52454 | 1.29181 | 0.70447 | 35 | 3UTR |
| hsa-let-7g-3p | NM_173799 | 2085 | 2106 | 1 | 0.00034 | 0.79366 | 0.98366 | 21 | 3UTR |
| hsa-let-7i-3p | NM_173799 | 2084 | 2106 | 1 | 0.00034 | 0.73721 | 0.99926 | 22 | 3UTR |
| hsa-miR-124-3p | NM_173799 | 2859 | 2877 | 1 | 0.00365 | 0 | 0 | 18 | 3UTR |
| hsa-miR-140-5p | NM_173799 | 1925 | 1944 | 1 | 0.00059 | 0.78574 | 0.24328 | 19 | 3UTR |
| hsa-miR-140-5p | NM_173799 | 971 | 991 | 1 | 0.01889 | 1.8302 | 1.90362 | 20 | 3UTR |
| hsa-miR-141-5p | NM_173799 | 1908 | 1928 | 1 | 2.45E-06 | 0.29669 | 0.38782 | 20 | 3UTR |
| hsa-miR-145-5p | NM_173799 | 2685 | 2706 | 1 | 0.00755 | 0 | 0 | 21 | 3UTR |
| hsa-miR-146a-3p | NM_173799 | 1828 | 1851 | 1 | 0.00087 | 0.12158 | 0.30445 | 23 | 3UTR |
| hsa-miR-150-5p | NM_173799 | 1898 | 1931 | 1 | 1.65E-06 | 0.30879 | 0.38262 | 22 | 3UTR |
| hsa-miR-193a-3p | NM_173799 | 1423 | 1455 | 1 | 0.00151 | 1.30405 | 0.31047 | 19 | 3UTR |
| hsa-miR-320a-5p | NM_173799 | 1821 | 1853 | 1 | 0.00205 | 0.24985 | 0.36018 | 26 | 3UTR |
| hsa-miR-320a-3p | NM_173799 | 1273 | 1295 | 1 | 0.03727 | 0.9047 | 0.88434 | 22 | 3UTR |
| hsa-miR-194-3p | NM_173799 | 1206 | 1243 | 1 | 0.00097 | 0.47481 | 0.60658 | 20 | 3UTR |
| hsa-miR-106b-3p | NM_173799 | 2715 | 2737 | 1 | 0.00016 | 0 | 0 | 22 | 3UTR |
| hsa-miR-30c-1-3p | NM_173799 | 894 | 920 | 1 | 0.11875 | 1.84195 | 1.84978 | 26 | 3UTR |
| hsa-miR-99b-5p | NM_173799 | 2020 | 2042 | 1 | 0.05757 | 0.50844 | 0.29111 | 22 | 3UTR |
| hsa-miR-99b-5p | NM_173799 | 2085 | 2110 | 1 | 0.00202 | 0.72335 | 1.10324 | 25 | 3UTR |
| hsa-miR-99b-5p | NM_173799 | 2465 | 2492 | 1 | 0.00126 | 0 | 0 | 27 | 3UTR |
| hsa-miR-99b-3p | NM_173799 | 2560 | 2586 | 1 | 0.00056 | 0 | 0 | 26 | 3UTR |
| hsa-miR-99b-3p | NM_173799 | 2684 | 2707 | 1 | 0.00077 | 0 | 0 | 23 | 3UTR |
| hsa-miR-365a-5p | NM_173799 | 1269 | 1285 | 1 | 6.00E-05 | 0.61637 | 0.87578 | 16 | 3UTR |
| hsa-miR-377-5p | NM_173799 | 771 | 788 | 1 | 3.28E-06 | 1.52685 | 1.64787 | 17 | 3UTR |
| hsa-miR-378a-5p | NM_173799 | 1351 | 1394 | 1 | 0.00366 | 0.21153 | 0.53317 | 43 | 3UTR |
| hsa-miR-382-5p | NM_173799 | 2684 | 2727 | 1 | 0.00013 | 0 | 0 | 26 | 3UTR |
| hsa-miR-328-3p | NM_173799 | 1818 | 1839 | 1 | 0.066 | 0.21502 | 0.30549 | 21 | 3UTR |
| hsa-miR-151a-3p | NM_173799 | 1465 | 1481 | 1 | 0.00027 | 0.08994 | 0.56131 | 16 | 3UTR |
| hsa-miR-148b-3p | NM_173799 | 2085 | 2109 | 1 | 0.00061 | 0.70601 | 1.00237 | 24 | 3UTR |
| hsa-miR-331-5p | NM_173799 | 2685 | 2708 | 1 | 0.00049 | 0 | 0 | 23 | 3UTR |
| hsa-miR-324-5p | NM_173799 | 1405 | 1445 | 1 | 0.00039 | -0.01882 | 0.38144 | 25 | 3UTR |
| hsa-miR-433-3p | NM_173799 | 1687 | 1707 | 1 | 0.00129 | 0.24998 | -0.0297 | 20 | 3UTR |
| hsa-miR-409-3p | NM_173799 | 2148 | 2174 | 1 | 0.00133 | 1.50671 | 1.48383 | 26 | 3UTR |
| hsa-miR-483-3p | NM_173799 | 1437 | 1472 | 1 | 0.00307 | 0.39951 | 0.52338 | 28 | 3UTR |
| hsa-miR-483-3p | NM_173799 | 1806 | 1845 | 1 | 0.01627 | 0.69224 | 0.4924 | 18 | 3UTR |
| hsa-miR-485-5p | NM_173799 | 960 | 976 | 1 | 6.84E-06 | 1.10147 | 1.99295 | 16 | 3UTR |
| hsa-miR-485-5p | NM_173799 | 1084 | 1099 | 1 | 0.00477 | 0.87306 | 1.46679 | 15 | 3UTR |
| hsa-miR-489-3p | NM_173799 | 833 | 871 | 1 | 0.03276 | 2.40531 | 1.48062 | 21 | 3UTR |
| hsa-miR-490-3p | NM_173799 | 1462 | 1482 | 1 | 0.00032 | 0.40101 | 0.50258 | 20 | 3UTR |
| hsa-miR-511-5p | NM_173799 | 1140 | 1162 | 1 | 0.01074 | 1.35707 | 1.31628 | 22 | 3UTR |
| hsa-miR-494-5p | NM_173799 | 1337 | 1362 | 1 | 0.00065 | -0.55586 | 1.11194 | 25 | 3UTR |
| hsa-miR-193b-3p | NM_173799 | 1818 | 1839 | 1 | 0.066 | 0.24221 | 0.27 | 21 | 3UTR |
| hsa-miR-512-3p | NM_173799 | 1587 | 1607 | 1 | 0.01342 | 1.55829 | 0.67388 | 20 | 3UTR |
| hsa-miR-515-5p | NM_173799 | 2405 | 2454 | 1 | 0.06355 | 0 | 0 | 26 | 3UTR |
| hsa-miR-515-3p | NM_173799 | 1510 | 1530 | 1 | 0.00034 | 0.39951 | 0.7889 | 20 | 3UTR |
| hsa-miR-523-3p | NM_173799 | 1231 | 1258 | 1 | 0.00034 | -0.22548 | 1.10215 | 27 | 3UTR |
| hsa-miR-518a-5p | NM_173799 | 1583 | 1596 | 1 | 0.0068 | 2.0423 | 0.85498 | 13 | 3UTR |
| hsa-miR-517c-3p | NM_173799 | 1429 | 1470 | 1 | 0.00231 | 1.17434 | 0.35819 | 21 | 3UTR |
| hsa-miR-527 | NM_173799 | 1583 | 1596 | 1 | 0.0068 | 2.0423 | 0.85498 | 13 | 3UTR |
| hsa-miR-499a-3p | NM_173799 | 1395 | 1417 | 1 | 0.00388 | 1.22507 | 0.24072 | 22 | 3UTR |
| hsa-miR-504-5p | NM_173799 | 967 | 981 | 1 | 0.00031 | 1.71725 | 1.94278 | 14 | 3UTR |
| hsa-miR-504-3p | NM_173799 | 2117 | 2137 | 1 | 0.00131 | 1.87644 | 0.98574 | 20 | 3UTR |
| hsa-miR-504-3p | NM_173799 | 1885 | 1906 | 1 | 0.12399 | 0.42525 | 0.43977 | 21 | 3UTR |
| hsa-miR-455-5p | NM_173799 | 772 | 783 | 1 | 6.01E-06 | 1.87746 | 1.60906 | 11 | 3UTR |
| hsa-miR-455-3p | NM_173799 | 878 | 900 | 1 | 0.00384 | 1.92809 | 2.02354 | 22 | 3UTR |
| hsa-miR-551a | NM_173799 | 1829 | 1854 | 1 | 0.002 | 0.25407 | 0.27 | 25 | 3UTR |
| hsa-miR-551a | NM_173799 | 2446 | 2491 | 1 | 0.00099 | 0 | 0 | 19 | 3UTR |
| hsa-miR-552-5p | NM_173799 | 2464 | 2479 | 1 | 0.00062 | 0 | 0 | 15 | 3UTR |
| hsa-miR-92b-3p | NM_173799 | 1829 | 1844 | 1 | 0.01573 | 0.12158 | 0.27624 | 15 | 3UTR |
| hsa-miR-567 | NM_173799 | 2682 | 2704 | 1 | 0.00083 | 0 | 0 | 16 | 3UTR |
| hsa-miR-571 | NM_173799 | 1065 | 1099 | 1 | 0.00477 | 1.85196 | 1.25143 | 20 | 3UTR |
| hsa-miR-588 | NM_173799 | 2722 | 2740 | 1 | 0.00028 | 0 | 0 | 18 | 3UTR |
| hsa-miR-550a-3p | NM_173799 | 1418 | 1451 | 1 | 0.00489 | 0.7973 | 0.62861 | 33 | 3UTR |
| hsa-miR-597-3p | NM_173799 | 1655 | 1701 | 1 | 0.00035 | 0.27548 | 0.19667 | 27 | 3UTR |
| hsa-miR-612 | NM_173799 | 1456 | 1481 | 1 | 0.00027 | 0.22481 | 0.47076 | 24 | 3UTR |
| hsa-miR-614 | NM_173799 | 1422 | 1474 | 1 | 0.00189 | 1.26462 | 0.30312 | 26 | 3UTR |
| hsa-miR-615-3p | NM_173799 | 2467 | 2490 | 1 | 0.00108 | 0 | 0 | 23 | 3UTR |
| hsa-miR-632 | NM_173799 | 1358 | 1377 | 1 | 0.00399 | 0.02624 | 0.42182 | 19 | 3UTR |
| hsa-miR-635 | NM_173799 | 959 | 979 | 1 | 0.00037 | 1.07506 | 1.97826 | 20 | 3UTR |
| hsa-miR-640 | NM_173799 | 966 | 983 | 1 | 1.15E-05 | 1.46312 | 1.95868 | 17 | 3UTR |
| hsa-miR-647 | NM_173799 | 2447 | 2468 | 1 | 0.00326 | 0 | 0 | 21 | 3UTR |
| hsa-miR-648 | NM_173799 | 2059 | 2107 | 1 | 0.0004 | 0.59163 | 0.60307 | 15 | 3UTR |
| hsa-miR-650 | NM_173799 | 1220 | 1239 | 1 | 0.00123 | 0.34788 | 0.78278 | 19 | 3UTR |
| hsa-miR-449b-3p | NM_173799 | 1104 | 1126 | 1 | 0.00507 | 1.91722 | 1.23308 | 22 | 3UTR |
| hsa-miR-449b-3p | NM_173799 | 2464 | 2491 | 1 | 0.00099 | 0 | 0 | 27 | 3UTR |
| hsa-miR-654-3p | NM_173799 | 1381 | 1402 | 1 | 0.00184 | 0.44576 | 0.11469 | 21 | 3UTR |
| hsa-miR-659-3p | NM_173799 | 1806 | 1840 | 1 | 0.01395 | 0.22244 | 0.35462 | 34 | 3UTR |
| hsa-miR-758-5p | NM_173799 | 1562 | 1580 | 1 | 0.00316 | 0.4108 | 1.25755 | 18 | 3UTR |
| hsa-miR-758-3p | NM_173799 | 2572 | 2594 | 1 | 0.00041 | 0 | 0 | 22 | 3UTR |
| hsa-miR-767-3p | NM_173799 | 1445 | 1469 | 1 | 0.00097 | 0.15209 | 0.56987 | 24 | 3UTR |
| hsa-miR-1224-3p | NM_173799 | 2008 | 2031 | 1 | 0.00036 | 0.12752 | 0.3691 | 23 | 3UTR |
| hsa-miR-320c | NM_173799 | 1013 | 1038 | 1 | 0.05792 | 1.4778 | 1.27223 | 17 | 3UTR |
| hsa-miR-1301-5p | NM_173799 | 967 | 982 | 1 | 0.00031 | 1.33097 | 1.94278 | 15 | 3UTR |
| hsa-miR-454-5p | NM_173799 | 1298 | 1325 | 1 | 0.06684 | 2.23682 | 0.5919 | 27 | 3UTR |
| hsa-miR-766-3p | NM_173799 | 1816 | 1843 | 1 | 0.01332 | 0.11713 | 0.27796 | 27 | 3UTR |
| hsa-miR-1298-5p | NM_173799 | 882 | 902 | 1 | 0.00051 | 1.45496 | 1.88037 | 20 | 3UTR |
| hsa-miR-765 | NM_173799 | 906 | 926 | 1 | 0.28926 | 2.29518 | 1.82409 | 20 | 3UTR |
| hsa-miR-675-5p | NM_173799 | 1708 | 1741 | 1 | 0.11798 | 0.02624 | 0.42793 | 33 | 3UTR |
| hsa-miR-892b | NM_173799 | 1667 | 1704 | 1 | 0.00278 | 0.68 | 0.08899 | 23 | 3UTR |
| hsa-miR-892b | NM_173799 | 1524 | 1547 | 1 | 0.00013 | 0.78456 | 1.00915 | 23 | 3UTR |
| hsa-miR-744-3p | NM_173799 | 1919 | 1935 | 1 | 4.06E-07 | 0.35131 | 0.42109 | 16 | 3UTR |
| hsa-miR-885-5p | NM_173799 | 1829 | 1852 | 1 | 0.00158 | 0.48037 | 0.22277 | 23 | 3UTR |
| hsa-miR-877-3p | NM_173799 | 1454 | 1484 | 1 | 0.00089 | 0.20891 | 0.56253 | 30 | 3UTR |
| hsa-miR-877-3p | NM_173799 | 1828 | 1852 | 1 | 0.00158 | 0.0845 | 0.3398 | 24 | 3UTR |
| hsa-miR-921 | NM_173799 | 1219 | 1243 | 1 | 0.00097 | 0.54676 | 0.83785 | 24 | 3UTR |
| hsa-miR-922 | NM_173799 | 1554 | 1573 | 1 | 0.00791 | 0.72079 | 1.08257 | 19 | 3UTR |
| hsa-miR-935 | NM_173799 | 2017 | 2046 | 1 | 0.05526 | 0.33513 | 0.35246 | 29 | 3UTR |
| hsa-miR-937-3p | NM_173799 | 2464 | 2491 | 1 | 0.00099 | 0 | 0 | 27 | 3UTR |
| hsa-miR-939-3p | NM_173799 | 1436 | 1483 | 1 | 0.00047 | 0.5017 | 0.54418 | 30 | 3UTR |
| hsa-miR-940 | NM_173799 | 1457 | 1479 | 1 | 0.00054 | 0.27484 | 0.55641 | 22 | 3UTR |
| hsa-miR-941 | NM_173799 | 1677 | 1717 | 1 | 0.00028 | 0.33902 | -0.08232 | 40 | 3UTR |
| hsa-miR-942-3p | NM_173799 | 1626 | 1656 | 1 | 5.47E-05 | 0.54785 | 0.67633 | 18 | 3UTR |
| hsa-miR-943 | NM_173799 | 2445 | 2477 | 1 | 0.00045 | 0 | 0 | 32 | 3UTR |
| hsa-miR-1178-3p | NM_173799 | 1423 | 1472 | 1 | 0.00307 | 0.8958 | 0.35574 | 19 | 3UTR |
| hsa-miR-1184 | NM_173799 | 1692 | 1716 | 1 | 0.00024 | 0.21502 | -0.04683 | 24 | 3UTR |
| hsa-miR-1233-3p | NM_173799 | 1833 | 1856 | 1 | 0.00369 | 0.26673 | 0.26609 | 23 | 3UTR |
| hsa-miR-1233-3p | NM_173799 | 1449 | 1474 | 1 | 0.00189 | 0.07363 | 0.62127 | 20 | 3UTR |
| hsa-miR-1234-3p | NM_173799 | 1380 | 1406 | 1 | 0.00042 | 0.64663 | 0.13059 | 26 | 3UTR |
| hsa-miR-1236-3p | NM_173799 | 1423 | 1463 | 1 | 0.00252 | 0.59814 | 0.50502 | 40 | 3UTR |
| hsa-miR-1237-3p | NM_173799 | 1436 | 1471 | 1 | 0.00351 | 0.39641 | 0.49401 | 35 | 3UTR |
| hsa-miR-1285-5p | NM_173799 | 1422 | 1443 | 1 | 0.00093 | 1.1341 | 0.35207 | 21 | 3UTR |
| hsa-miR-1289 | NM_173799 | 1785 | 1815 | 1 | 0.00565 | 0.29772 | 0.2376 | 30 | 3UTR |
| hsa-miR-1294 | NM_173799 | 1141 | 1162 | 1 | 0.01074 | -0.12759 | 1.3481 | 21 | 3UTR |
| hsa-miR-1303 | NM_173799 | 1051 | 1074 | 1 | 0.00025 | 1.23135 | 1.22941 | 23 | 3UTR |
| hsa-miR-1247-5p | NM_173799 | 2005 | 2027 | 1 | 0.00018 | 0.15905 | 0.38158 | 22 | 3UTR |
| hsa-miR-1250-5p | NM_173799 | 1520 | 1536 | 1 | 0.00397 | 1.53654 | 0.91249 | 16 | 3UTR |
| hsa-miR-1251-5p | NM_173799 | 1908 | 1932 | 1 | 1.76E-06 | 0.2866 | 0.39406 | 18 | 3UTR |
| hsa-miR-1265 | NM_173799 | 1043 | 1063 | 1 | 0.0293 | 1.3016 | 1.29793 | 20 | 3UTR |
| hsa-miR-1266-5p | NM_173799 | 1219 | 1245 | 1 | 3.50E-05 | -0.57136 | 0.96388 | 26 | 3UTR |
| hsa-miR-1272 | NM_173799 | 1129 | 1163 | 1 | 0.0131 | 2.20545 | 0.99325 | 20 | 3UTR |
| hsa-miR-1281 | NM_173799 | 2447 | 2458 | 1 | 0.08666 | 0 | 0 | 11 | 3UTR |
| hsa-miR-1288-5p | NM_173799 | 977 | 1009 | 1 | 0.11686 | 1.64294 | 1.50839 | 32 | 3UTR |
| hsa-miR-1288-3p | NM_173799 | 1550 | 1575 | 1 | 0.00492 | 0.86858 | 1.13396 | 25 | 3UTR |
| hsa-miR-1292-3p | NM_173799 | 1807 | 1845 | 1 | 0.01627 | 0.53806 | 0.4582 | 20 | 3UTR |
| hsa-miR-664a-5p | NM_173799 | 909 | 940 | 1 | 0.10954 | 2.18506 | 1.81552 | 23 | 3UTR |
| hsa-miR-664a-3p | NM_173799 | 1921 | 1939 | 1 | 1.56E-05 | 0.24963 | 0.43669 | 18 | 3UTR |
| hsa-miR-1306-5p | NM_173799 | 2017 | 2031 | 1 | 0.00036 | 0.30425 | 0.3327 | 14 | 3UTR |
| hsa-miR-1321 | NM_173799 | 920 | 937 | 1 | 0.1118 | 2.22176 | 1.78126 | 17 | 3UTR |
| hsa-miR-1197 | NM_173799 | 1628 | 1661 | 1 | 1.66E-07 | 0.69468 | 0.84886 | 16 | 3UTR |
| hsa-miR-1324 | NM_173799 | 1445 | 1482 | 1 | 0.00032 | 0.18566 | 0.51848 | 27 | 3UTR |
| hsa-miR-1470 | NM_173799 | 1829 | 1853 | 1 | 0.00205 | 0.39303 | 0.23011 | 24 | 3UTR |
| hsa-miR-1471 | NM_173799 | 2712 | 2739 | 1 | 0.00024 | 0 | 0 | 27 | 3UTR |
| hsa-miR-320d | NM_173799 | 1013 | 1038 | 1 | 0.05792 | 1.4778 | 1.27223 | 17 | 3UTR |
| hsa-miR-1825 | NM_173799 | 2019 | 2040 | 1 | 0.02851 | 0.17568 | 0.31087 | 21 | 3UTR |
| hsa-miR-1909-5p | NM_173799 | 1568 | 1609 | 1 | 0.01308 | 0.61103 | 1.04586 | 41 | 3UTR |
| hsa-miR-1910-3p | NM_173799 | 1262 | 1281 | 1 | 3.51E-05 | 1.62553 | 0.90025 | 19 | 3UTR |
| hsa-miR-1910-3p | NM_173799 | 2122 | 2135 | 1 | 0.00053 | 1.99198 | 0.93894 | 13 | 3UTR |
| hsa-miR-1976 | NM_173799 | 1328 | 1348 | 1 | 0.20911 | 0.37076 | 0.82806 | 20 | 3UTR |
| hsa-miR-2276-5p | NM_173799 | 1811 | 1853 | 1 | 0.00205 | 0.36186 | 0.43745 | 20 | 3UTR |
| hsa-miR-2682-5p | NM_173799 | 1043 | 1069 | 1 | 0.00087 | 1.02405 | 1.39092 | 26 | 3UTR |
| hsa-miR-2909 | NM_173799 | 1513 | 1533 | 1 | 0.00513 | 0.18783 | 0.85008 | 11 | 3UTR |
| hsa-miR-3124-3p | NM_173799 | 1812 | 1853 | 1 | 0.00205 | 0.63377 | 0.46475 | 19 | 3UTR |
| hsa-miR-3126-5p | NM_173799 | 1232 | 1282 | 1 | 6.21E-05 | -0.28258 | 0.96877 | 20 | 3UTR |
| hsa-miR-3127-5p | NM_173799 | 1511 | 1532 | 1 | 0.00509 | 0.56851 | 0.7681 | 21 | 3UTR |
| hsa-miR-3130-5p | NM_173799 | 2470 | 2492 | 1 | 0.00126 | 0 | 0 | 22 | 3UTR |
| hsa-miR-3130-3p | NM_173799 | 1219 | 1235 | 1 | 0.00094 | -0.42126 | 0.90637 | 16 | 3UTR |
| hsa-miR-3141 | NM_173799 | 910 | 943 | 1 | 0.09227 | 2.19117 | 1.52063 | 33 | 3UTR |
| hsa-miR-3147 | NM_173799 | 1054 | 1099 | 1 | 0.00477 | 1.12866 | 1.18291 | 25 | 3UTR |
| hsa-miR-3151-3p | NM_173799 | 1827 | 1851 | 1 | 0.00087 | 0.10082 | 0.26315 | 24 | 3UTR |
| hsa-miR-3155a | NM_173799 | 1140 | 1154 | 1 | 5.05E-05 | 0.82461 | 1.2759 | 14 | 3UTR |
| hsa-miR-3157-3p | NM_173799 | 2129 | 2156 | 1 | 0.00045 | 2.0613 | 1.01827 | 27 | 3UTR |
| hsa-miR-3162-5p | NM_173799 | 1881 | 1909 | 1 | 0.07589 | 0.48863 | 0.50344 | 28 | 3UTR |
| hsa-miR-3162-3p | NM_173799 | 1819 | 1841 | 1 | 0.01091 | 0.0845 | 0.3398 | 22 | 3UTR |
| hsa-miR-3170 | NM_173799 | 2263 | 2280 | 1 | 0.00458 | 0 | 0 | 17 | 3UTR |
| hsa-miR-3173-5p | NM_173799 | 1818 | 1836 | 1 | 0.04149 | 0.39449 | 0.47789 | 18 | 3UTR |
| hsa-miR-3173-3p | NM_173799 | 1629 | 1647 | 1 | 8.76E-05 | 1.01912 | 0.82194 | 18 | 3UTR |
| hsa-miR-323b-5p | NM_173799 | 1603 | 1624 | 1 | 0.00194 | 2.23155 | 0.47688 | 21 | 3UTR |
| hsa-miR-323b-5p | NM_173799 | 2700 | 2722 | 1 | 0.00022 | 0 | 0 | 22 | 3UTR |
| hsa-miR-3180-5p | NM_173799 | 1439 | 1462 | 1 | 0.00252 | 0.02468 | 0.58211 | 23 | 3UTR |
| hsa-miR-3184-3p | NM_173799 | 1828 | 1855 | 1 | 0.00211 | 0.49141 | 0.22075 | 27 | 3UTR |
| hsa-miR-3189-3p | NM_173799 | 947 | 980 | 1 | 0.00041 | 1.65584 | 1.88771 | 33 | 3UTR |
| hsa-miR-3190-5p | NM_173799 | 1380 | 1404 | 1 | 0.00053 | 0.87228 | 0.17954 | 24 | 3UTR |
| hsa-miR-3190-5p | NM_173799 | 1436 | 1454 | 1 | 0.0041 | 1.23063 | 0.43283 | 18 | 3UTR |
| hsa-miR-3190-3p | NM_173799 | 1563 | 1596 | 1 | 0.0068 | 1.90362 | 0.84397 | 33 | 3UTR |
| hsa-miR-3190-3p | NM_173799 | 1961 | 1998 | 1 | 0.00171 | 0.25887 | 0.40861 | 37 | 3UTR |
| hsa-miR-3198 | NM_173799 | 922 | 957 | 1 | 0.21082 | 2.27071 | 1.79717 | 35 | 3UTR |
| hsa-miR-3202 | NM_173799 | 912 | 928 | 1 | 0.37618 | 2.3066 | 1.77637 | 16 | 3UTR |
| hsa-miR-4301 | NM_173799 | 1352 | 1398 | 1 | 0.00291 | -0.09496 | 0.75709 | 17 | 3UTR |
| hsa-miR-4304 | NM_173799 | 1220 | 1236 | 1 | 0.00106 | 0.07635 | 0.82806 | 16 | 3UTR |
| hsa-miR-4303 | NM_173799 | 2264 | 2278 | 1 | 0.00731 | 0 | 0 | 14 | 3UTR |
| hsa-miR-4305 | NM_173799 | 1447 | 1482 | 1 | 0.00032 | 0.09266 | 0.68979 | 22 | 3UTR |
| hsa-miR-4313 | NM_173799 | 1806 | 1837 | 1 | 0.04968 | 0.60658 | 0.49356 | 19 | 3UTR |
| hsa-miR-4313 | NM_173799 | 1377 | 1399 | 1 | 0.0027 | 0.35098 | 0.1514 | 22 | 3UTR |
| hsa-miR-4323 | NM_173799 | 1816 | 1838 | 1 | 0.05672 | -0.05767 | 0.27172 | 22 | 3UTR |
| hsa-miR-4258 | NM_173799 | 1826 | 1840 | 1 | 0.01395 | 0.19055 | 0.39951 | 14 | 3UTR |
| hsa-miR-4265 | NM_173799 | 1497 | 1519 | 1 | 0.02979 | 0.24998 | 0.68 | 22 | 3UTR |
| hsa-miR-4268 | NM_173799 | 1396 | 1466 | 1 | 0.00031 | 0.48024 | 0.2554 | 45 | 3UTR |
| hsa-miR-4270 | NM_173799 | 1888 | 1909 | 1 | 0.07589 | 0.76725 | 0.40445 | 21 | 3UTR |
| hsa-miR-4279 | NM_173799 | 1830 | 1852 | 1 | 0.00158 | 0.26107 | 0.29251 | 22 | 3UTR |
| hsa-miR-4292 | NM_173799 | 962 | 980 | 1 | 0.00041 | 1.41689 | 1.98805 | 18 | 3UTR |
| hsa-miR-4290 | NM_173799 | 1805 | 1836 | 1 | 0.04149 | 0.61637 | 0.46181 | 19 | 3UTR |
| hsa-miR-500b-5p | NM_173799 | 1421 | 1443 | 1 | 0.00093 | 1.2988 | 0.36431 | 22 | 3UTR |
| hsa-miR-3605-5p | NM_173799 | 1112 | 1161 | 1 | 0.00768 | 2.46105 | 1.06544 | 26 | 3UTR |
| hsa-miR-3614-5p | NM_173799 | 1043 | 1062 | 1 | 0.01884 | 1.07156 | 1.32485 | 19 | 3UTR |
| hsa-miR-3616-3p | NM_173799 | 1731 | 1760 | 1 | 0.01118 | 0.02624 | 0.50625 | 29 | 3UTR |
| hsa-miR-3619-3p | NM_173799 | 952 | 988 | 1 | 0.00044 | 1.65222 | 1.8559 | 27 | 3UTR |
| hsa-miR-3659 | NM_173799 | 1220 | 1238 | 1 | 0.00109 | 0.12692 | 0.75097 | 18 | 3UTR |
| hsa-miR-3659 | NM_173799 | 1357 | 1381 | 1 | 0.01653 | 0.26397 | 0.20523 | 24 | 3UTR |
| hsa-miR-3663-5p | NM_173799 | 1526 | 1548 | 1 | 0.00018 | 1.24287 | 0.877 | 22 | 3UTR |
| hsa-miR-3675-5p | NM_173799 | 2259 | 2280 | 1 | 0.00458 | 0 | 0 | 21 | 3UTR |
| hsa-miR-3675-3p | NM_173799 | 1455 | 1484 | 1 | 0.00089 | 0.30003 | 0.56253 | 29 | 3UTR |
| hsa-miR-3680-3p | NM_173799 | 2126 | 2155 | 1 | 0.00061 | 1.99693 | 0.95613 | 29 | 3UTR |
| hsa-miR-3690 | NM_173799 | 1911 | 1932 | 1 | 1.76E-06 | 0.46223 | 0.42109 | 21 | 3UTR |
| hsa-miR-3922-3p | NM_173799 | 1425 | 1454 | 1 | 0.0041 | 1.25177 | 0.46709 | 21 | 3UTR |
| hsa-miR-676-5p | NM_173799 | 1905 | 1931 | 1 | 1.65E-06 | 0.32127 | 0.38366 | 17 | 3UTR |
| hsa-miR-3928-5p | NM_173799 | 1963 | 1987 | 1 | 0.0189 | 0.11207 | 0.44501 | 24 | 3UTR |
| hsa-miR-3939 | NM_173799 | 844 | 862 | 1 | 0.00089 | 0.74254 | 1.90816 | 18 | 3UTR |
| hsa-miR-3940-5p | NM_173799 | 1146 | 1167 | 1 | 0.08534 | 0.43178 | 1.364 | 21 | 3UTR |
| hsa-miR-3944-5p | NM_173799 | 2089 | 2106 | 1 | 0.00034 | 0.44189 | 1.12716 | 17 | 3UTR |
| hsa-miR-378e | NM_173799 | 2257 | 2285 | 1 | 0.00149 | 0 | 0 | 19 | 3UTR |
| hsa-miR-4421 | NM_173799 | 2716 | 2758 | 1 | 0.00077 | 0 | 0 | 42 | 3UTR |
| hsa-miR-4423-5p | NM_173799 | 1819 | 1837 | 1 | 0.04968 | 0.12413 | 0.40233 | 18 | 3UTR |
| hsa-miR-4425 | NM_173799 | 2124 | 2141 | 1 | 0.00282 | 1.65229 | 0.98158 | 17 | 3UTR |
| hsa-miR-4428 | NM_173799 | 917 | 937 | 1 | 0.1118 | 2.24624 | 1.747 | 20 | 3UTR |
| hsa-miR-4430 | NM_173799 | 1938 | 1955 | 1 | 0.02381 | 1.5294 | 0.3587 | 17 | 3UTR |
| hsa-miR-4432 | NM_173799 | 776 | 809 | 1 | 0.00267 | 1.51147 | 1.96651 | 33 | 3UTR |
| hsa-miR-4445-3p | NM_173799 | 1555 | 1600 | 1 | 0.0036 | 0.51481 | 1.15477 | 18 | 3UTR |
| hsa-miR-4446-5p | NM_173799 | 1533 | 1576 | 1 | 0.00417 | 0.81168 | 1.06422 | 43 | 3UTR |
| hsa-miR-4448 | NM_173799 | 1515 | 1545 | 1 | 0.00014 | 0.68129 | 0.9027 | 23 | 3UTR |
| hsa-miR-4450 | NM_173799 | 907 | 929 | 1 | 0.44358 | 2.13611 | 1.91463 | 22 | 3UTR |
| hsa-miR-4450 | NM_173799 | 2263 | 2287 | 1 | 0.0009 | 0 | 0 | 24 | 3UTR |
| hsa-miR-4454 | NM_173799 | 1592 | 1612 | 1 | 0.04559 | 1.61267 | 0.64941 | 20 | 3UTR |
| hsa-miR-4456 | NM_173799 | 1659 | 1674 | 1 | 1.28E-07 | -0.08408 | 0.47566 | 15 | 3UTR |
| hsa-miR-4472 | NM_173799 | 2694 | 2714 | 1 | 0.04068 | 0 | 0 | 20 | 3UTR |
| hsa-miR-4478 | NM_173799 | 1221 | 1238 | 1 | 0.00109 | -0.25345 | 0.78645 | 17 | 3UTR |
| hsa-miR-3155b | NM_173799 | 1140 | 1154 | 1 | 5.05E-05 | 0.82461 | 1.2759 | 14 | 3UTR |
| hsa-miR-4485-5p | NM_173799 | 1542 | 1577 | 1 | 0.00405 | 1.20371 | 0.8611 | 15 | 3UTR |
| hsa-miR-4494 | NM_173799 | 1359 | 1384 | 1 | 0.01003 | 0.32923 | 0.16485 | 25 | 3UTR |
| hsa-miR-4496 | NM_173799 | 913 | 936 | 1 | 0.15048 | 1.8302 | 1.85223 | 23 | 3UTR |
| hsa-miR-4498 | NM_173799 | 2100 | 2138 | 1 | 0.00169 | 0.8204 | 1.03253 | 21 | 3UTR |
| hsa-miR-4505 | NM_173799 | 1220 | 1237 | 1 | 0.00108 | 0.36186 | 0.82561 | 17 | 3UTR |
| hsa-miR-4510 | NM_173799 | 2100 | 2137 | 1 | 0.00131 | 0.86679 | 0.99302 | 21 | 3UTR |
| hsa-miR-4516 | NM_173799 | 1524 | 1569 | 1 | 0.00126 | 0.81571 | 1.28569 | 45 | 3UTR |
| hsa-miR-4524a-3p | NM_173799 | 1042 | 1070 | 1 | 0.00092 | 0.9492 | 1.29181 | 28 | 3UTR |
| hsa-miR-4527 | NM_173799 | 1563 | 1577 | 1 | 0.00405 | 0.00293 | 1.10337 | 14 | 3UTR |
| hsa-miR-4534 | NM_173799 | 911 | 926 | 1 | 0.28926 | 2.20778 | 1.78126 | 15 | 3UTR |
| hsa-miR-4536-3p | NM_173799 | 856 | 878 | 1 | 0.25115 | 1.10048 | 1.99448 | 22 | 3UTR |
| hsa-miR-4639-3p | NM_173799 | 1825 | 1845 | 1 | 0.01627 | 0.10173 | 0.27588 | 20 | 3UTR |
| hsa-miR-4640-3p | NM_173799 | 1831 | 1853 | 1 | 0.00205 | 0.37217 | 0.26315 | 22 | 3UTR |
| hsa-miR-4642 | NM_173799 | 1420 | 1453 | 1 | 0.00534 | 0.76973 | 0.38144 | 26 | 3UTR |
| hsa-miR-4650-3p | NM_173799 | 1681 | 1730 | 1 | 0.01333 | 0.37165 | 0.05473 | 20 | 3UTR |
| hsa-miR-4652-3p | NM_173799 | 1828 | 1850 | 1 | 0.00112 | 0.64489 | 0.1909 | 22 | 3UTR |
| hsa-miR-4656 | NM_173799 | 1626 | 1651 | 1 | 2.56E-05 | 0.05313 | 0.76076 | 25 | 3UTR |
| hsa-miR-4657 | NM_173799 | 2711 | 2733 | 1 | 3.30E-05 | 0 | 0 | 22 | 3UTR |
| hsa-miR-4660 | NM_173799 | 834 | 858 | 1 | 0.00025 | 2.71121 | 1.52072 | 17 | 3UTR |
| hsa-miR-4659b-3p | NM_173799 | 1445 | 1465 | 1 | 0.00023 | 0.06819 | 0.60658 | 20 | 3UTR |
| hsa-miR-4667-5p | NM_173799 | 2648 | 2667 | 1 | 2.42E-05 | 0 | 0 | 19 | 3UTR |
| hsa-miR-4667-3p | NM_173799 | 1819 | 1841 | 1 | 0.01091 | 0.12843 | 0.29704 | 22 | 3UTR |
| hsa-miR-4685-3p | NM_173799 | 1523 | 1543 | 1 | 2.25E-05 | 0.4178 | 0.89658 | 20 | 3UTR |
| hsa-miR-4691-3p | NM_173799 | 835 | 856 | 1 | 0.00015 | 2.40667 | 1.52439 | 21 | 3UTR |
| hsa-miR-4697-5p | NM_173799 | 1134 | 1159 | 1 | 0.00311 | 0.98727 | 1.22574 | 25 | 3UTR |
| hsa-miR-4700-5p | NM_173799 | 2119 | 2141 | 1 | 0.00282 | 1.8561 | 0.9327 | 22 | 3UTR |
| hsa-miR-4707-5p | NM_173799 | 1627 | 1664 | 1 | 3.89E-08 | 0.18566 | 0.69835 | 21 | 3UTR |
| hsa-miR-4707-3p | NM_173799 | 1885 | 1932 | 1 | 1.76E-06 | 0.50844 | 0.51066 | 24 | 3UTR |
| hsa-miR-4708-5p | NM_173799 | 1381 | 1401 | 1 | 0.0015 | 0.76372 | 0.16853 | 20 | 3UTR |
| hsa-miR-4713-5p | NM_173799 | 1828 | 1852 | 1 | 0.00158 | 0.12692 | 0.34084 | 24 | 3UTR |
| hsa-miR-4717-5p | NM_173799 | 2530 | 2568 | 1 | 0.09502 | 0 | 0 | 38 | 3UTR |
| hsa-miR-4717-3p | NM_173799 | 1637 | 1657 | 1 | 4.65E-05 | -0.89847 | 0.66042 | 20 | 3UTR |
| hsa-miR-4722-3p | NM_173799 | 2017 | 2041 | 1 | 0.04083 | 0.17569 | 0.3743 | 24 | 3UTR |
| hsa-miR-4725-5p | NM_173799 | 1820 | 1855 | 1 | 0.00211 | 0.30748 | 0.4142 | 28 | 3UTR |
| hsa-miR-4727-3p | NM_173799 | 2718 | 2731 | 1 | 0.00202 | 0 | 0 | 13 | 3UTR |
| hsa-miR-4728-5p | NM_173799 | 1630 | 1651 | 1 | 2.56E-05 | 0.26397 | 0.69468 | 21 | 3UTR |
| hsa-miR-4732-3p | NM_173799 | 1460 | 1483 | 1 | 0.00047 | 0.13345 | 0.61637 | 23 | 3UTR |
| hsa-miR-4734 | NM_173799 | 1617 | 1640 | 1 | 1.15E-05 | 0.69468 | 0.84886 | 23 | 3UTR |
| hsa-miR-3064-5p | NM_173799 | 1076 | 1098 | 1 | 0.00662 | 1.03776 | 1.37502 | 22 | 3UTR |
| hsa-miR-4738-5p | NM_173799 | 2064 | 2110 | 1 | 0.00202 | 0.57684 | 1.06061 | 46 | 3UTR |
| hsa-miR-4740-5p | NM_173799 | 1422 | 1468 | 1 | 0.00118 | 0.74643 | 0.37899 | 20 | 3UTR |
| hsa-miR-4740-5p | NM_173799 | 2263 | 2286 | 1 | 0.00117 | 0 | 0 | 23 | 3UTR |
| hsa-miR-4743-5p | NM_173799 | 1619 | 1652 | 1 | 0.00024 | 0.32181 | 0.7473 | 33 | 3UTR |
| hsa-miR-4743-3p | NM_173799 | 1831 | 1851 | 1 | 0.00087 | 0.15629 | 0.26419 | 20 | 3UTR |
| hsa-miR-4747-3p | NM_173799 | 1692 | 1721 | 1 | 0.00088 | 0.4411 | 0.03637 | 29 | 3UTR |
| hsa-miR-4753-5p | NM_173799 | 912 | 942 | 1 | 0.10339 | 2.1938 | 1.85101 | 19 | 3UTR |
| hsa-miR-4753-3p | NM_173799 | 1429 | 1465 | 1 | 0.00023 | 0.66112 | 0.61637 | 36 | 3UTR |
| hsa-miR-4769-5p | NM_173799 | 1722 | 1745 | 1 | 0.09394 | -0.0297 | 0.39734 | 23 | 3UTR |
| hsa-miR-4769-3p | NM_173799 | 1808 | 1842 | 1 | 0.01144 | 0.41944 | 0.44504 | 26 | 3UTR |
| hsa-miR-4777-3p | NM_173799 | 1374 | 1393 | 1 | 0.00382 | 0.29334 | 0.10979 | 19 | 3UTR |
| hsa-miR-4783-5p | NM_173799 | 1420 | 1456 | 1 | 0.00121 | 0.71752 | 0.62616 | 36 | 3UTR |
| hsa-miR-4783-3p | NM_173799 | 1519 | 1541 | 1 | 2.44E-05 | 0.67333 | 0.9027 | 22 | 3UTR |
| hsa-miR-2467-5p | NM_173799 | 1694 | 1721 | 1 | 0.00088 | 0.37338 | 0.09756 | 27 | 3UTR |
| hsa-miR-2467-3p | NM_173799 | 1710 | 1737 | 1 | 0.19041 | 0.02903 | 0.20891 | 27 | 3UTR |
| hsa-miR-4786-5p | NM_173799 | 1928 | 1978 | 1 | 0.00092 | 0.73952 | 0.22768 | 22 | 3UTR |
| hsa-miR-4787-5p | NM_173799 | 1437 | 1459 | 1 | 0.0011 | 1.17761 | 0.47198 | 22 | 3UTR |
| hsa-miR-4794 | NM_173799 | 1374 | 1398 | 1 | 0.00291 | 0.42963 | 0.09144 | 24 | 3UTR |
| hsa-miR-4794 | NM_173799 | 1053 | 1098 | 1 | 0.00662 | 1.24287 | 1.43375 | 35 | 3UTR |
| hsa-miR-4796-5p | NM_173799 | 2464 | 2479 | 1 | 0.00062 | 0 | 0 | 15 | 3UTR |
| hsa-miR-4796-5p | NM_173799 | 1837 | 1850 | 1 | 0.00112 | 0.50976 | 0.21659 | 13 | 3UTR |
| hsa-miR-4804-5p | NM_173799 | 932 | 949 | 1 | 0.07761 | 2.34141 | 1.69438 | 17 | 3UTR |
| hsa-miR-5002-3p | NM_173799 | 1368 | 1396 | 1 | 0.00582 | 0.36186 | 0.25051 | 17 | 3UTR |
| hsa-miR-5004-5p | NM_173799 | 2122 | 2135 | 1 | 0.00053 | 2.08903 | 0.98886 | 13 | 3UTR |
| hsa-miR-5006-5p | NM_173799 | 1513 | 1534 | 1 | 0.00521 | 0.34011 | 0.88067 | 21 | 3UTR |
| hsa-miR-5008-3p | NM_173799 | 1444 | 1469 | 1 | 0.00097 | 0.20063 | 0.55641 | 25 | 3UTR |
| hsa-miR-5087 | NM_173799 | 1078 | 1116 | 1 | 6.29E-05 | 0.94454 | 1.4154 | 25 | 3UTR |
| hsa-miR-5088-5p | NM_173799 | 916 | 942 | 1 | 0.10339 | 2.41754 | 1.84734 | 26 | 3UTR |
| hsa-miR-5093 | NM_173799 | 912 | 935 | 1 | 0.4227 | 2.20397 | 1.82531 | 23 | 3UTR |
| hsa-miR-5093 | NM_173799 | 1221 | 1238 | 1 | 0.00109 | -0.24995 | 0.77177 | 17 | 3UTR |
| hsa-miR-5192 | NM_173799 | 2671 | 2729 | 1 | 0.0002 | 0 | 0 | 22 | 3UTR |
| hsa-miR-5196-3p | NM_173799 | 1806 | 1845 | 1 | 0.01627 | 0.43364 | 0.42682 | 26 | 3UTR |
| hsa-miR-4524b-3p | NM_173799 | 1042 | 1070 | 1 | 0.00092 | 0.9492 | 1.29181 | 28 | 3UTR |
| hsa-miR-664b-5p | NM_173799 | 918 | 941 | 1 | 0.10647 | 2.18506 | 1.80206 | 23 | 3UTR |
| hsa-miR-664b-3p | NM_173799 | 1831 | 1852 | 1 | 0.00158 | 0.20196 | 0.27251 | 21 | 3UTR |
| hsa-miR-5588-5p | NM_173799 | 1506 | 1528 | 1 | 0.00094 | 0.52204 | 0.80359 | 22 | 3UTR |
| hsa-miR-5684 | NM_173799 | 1904 | 1932 | 1 | 1.76E-06 | 0.15905 | 0.3951 | 20 | 3UTR |
| hsa-miR-5691 | NM_173799 | 1495 | 1513 | 1 | 0.00017 | 0.60213 | 0.54418 | 18 | 3UTR |
| hsa-miR-5691 | NM_173799 | 1811 | 1845 | 1 | 0.01627 | 0.45975 | 0.46829 | 16 | 3UTR |
| hsa-miR-5704 | NM_173799 | 2525 | 2568 | 1 | 0.09502 | 0 | 0 | 35 | 3UTR |
| hsa-miR-5739 | NM_173799 | 1991 | 2008 | 1 | 0.00114 | 0.52693 | 0.3847 | 17 | 3UTR |
| hsa-miR-6075 | NM_173799 | 1696 | 1719 | 1 | 0.00032 | 0.45975 | -0.00768 | 23 | 3UTR |
| hsa-miR-6077 | NM_173799 | 1615 | 1647 | 1 | 8.76E-05 | 0.4974 | 0.76932 | 20 | 3UTR |
| hsa-miR-6080 | NM_173799 | 1436 | 1454 | 1 | 0.0041 | 1.19936 | 0.46587 | 18 | 3UTR |
| hsa-miR-6086 | NM_173799 | 1220 | 1239 | 1 | 0.00123 | -0.38455 | 0.77911 | 19 | 3UTR |
| hsa-miR-6127 | NM_173799 | 1989 | 2009 | 1 | 0.00086 | 0.53617 | 0.37742 | 20 | 3UTR |
| hsa-miR-378j | NM_173799 | 1021 | 1046 | 1 | 0.0015 | 0.94308 | 1.37502 | 18 | 3UTR |
| hsa-miR-378j | NM_173799 | 2263 | 2285 | 1 | 0.00149 | 0 | 0 | 22 | 3UTR |
| hsa-miR-6130 | NM_173799 | 1895 | 1909 | 1 | 0.07589 | 0.54625 | 0.38262 | 14 | 3UTR |
| hsa-miR-6133 | NM_173799 | 1630 | 1647 | 1 | 8.76E-05 | 0.46587 | 0.9027 | 17 | 3UTR |
| hsa-miR-6165 | NM_173799 | 1900 | 1922 | 1 | 0.00197 | 0.46685 | 0.37118 | 22 | 3UTR |
| hsa-miR-6501-5p | NM_173799 | 1172 | 1232 | 1 | 0.00483 | 0.87578 | 0.68734 | 34 | 3UTR |
| hsa-miR-6501-5p | NM_173799 | 1516 | 1536 | 1 | 0.00397 | 0.3227 | 0.86477 | 20 | 3UTR |
| hsa-miR-6503-5p | NM_173799 | 1040 | 1063 | 1 | 0.0293 | 0.79692 | 1.20493 | 23 | 3UTR |
| hsa-miR-6504-5p | NM_173799 | 957 | 979 | 1 | 0.00037 | 1.40099 | 1.93666 | 22 | 3UTR |
| hsa-miR-6505-3p | NM_173799 | 2444 | 2475 | 1 | 0.00084 | 0 | 0 | 31 | 3UTR |
| hsa-miR-6510-3p | NM_173799 | 2465 | 2489 | 1 | 0.0017 | 0 | 0 | 24 | 3UTR |
| hsa-miR-6511a-3p | NM_173799 | 1421 | 1450 | 1 | 0.00458 | 1.23634 | 0.41937 | 29 | 3UTR |
| hsa-miR-6514-5p | NM_173799 | 1502 | 1518 | 1 | 0.02426 | 0.26397 | 0.6592 | 16 | 3UTR |
| hsa-miR-6515-3p | NM_173799 | 1816 | 1851 | 1 | 0.00087 | 0.38856 | 0.38031 | 24 | 3UTR |
| hsa-miR-6716-3p | NM_173799 | 2006 | 2032 | 1 | 0.00043 | 0.03996 | 0.36078 | 26 | 3UTR |
| hsa-miR-6716-3p | NM_173799 | 2465 | 2490 | 1 | 0.00108 | 0 | 0 | 25 | 3UTR |
| hsa-miR-6716-3p | NM_173799 | 1817 | 1841 | 1 | 0.01091 | 0.76565 | 0.46737 | 24 | 3UTR |
| hsa-miR-6721-5p | NM_173799 | 1511 | 1537 | 1 | 0.00371 | 0.20803 | 0.77177 | 26 | 3UTR |
| hsa-miR-6722-5p | NM_173799 | 854 | 875 | 1 | 0.16796 | 0.71426 | 1.89547 | 21 | 3UTR |
| hsa-miR-892c-3p | NM_173799 | 1358 | 1380 | 1 | 0.0213 | 0.05273 | 0.40101 | 22 | 3UTR |
| hsa-miR-6727-5p | NM_173799 | 1622 | 1663 | 1 | 2.37E-08 | 1.26066 | 0.8097 | 23 | 3UTR |
| hsa-miR-6727-3p | NM_173799 | 1454 | 1470 | 1 | 0.00231 | 0.37817 | 0.5662 | 16 | 3UTR |
| hsa-miR-6728-3p | NM_173799 | 1454 | 1484 | 1 | 0.00089 | 0.39165 | 0.51481 | 30 | 3UTR |
| hsa-miR-6731-5p | NM_173799 | 2119 | 2138 | 1 | 0.00169 | 1.80249 | 0.95766 | 19 | 3UTR |
| hsa-miR-6731-3p | NM_173799 | 1828 | 1849 | 1 | 0.00075 | 0.46338 | 0.23782 | 21 | 3UTR |
| hsa-miR-6732-3p | NM_173799 | 1454 | 1483 | 1 | 0.00047 | 0.30312 | 0.55397 | 29 | 3UTR |
| hsa-miR-6734-3p | NM_173799 | 1828 | 1852 | 1 | 0.00158 | 0.10366 | 0.31381 | 24 | 3UTR |
| hsa-miR-6735-3p | NM_173799 | 1379 | 1407 | 1 | 0.00153 | 0.84043 | 0.15751 | 28 | 3UTR |
| hsa-miR-6737-3p | NM_173799 | 1423 | 1469 | 1 | 0.00097 | 0.46568 | 0.5662 | 46 | 3UTR |
| hsa-miR-6739-3p | NM_173799 | 1454 | 1470 | 1 | 0.00231 | 0.41624 | 0.58089 | 16 | 3UTR |
| hsa-miR-6740-5p | NM_173799 | 938 | 956 | 1 | 0.2124 | 2.17282 | 1.80818 | 18 | 3UTR |
| hsa-miR-6740-3p | NM_173799 | 1441 | 1471 | 1 | 0.00351 | 0.15955 | 0.59068 | 23 | 3UTR |
| hsa-miR-6741-3p | NM_173799 | 1816 | 1839 | 1 | 0.066 | 0.21502 | 0.30549 | 23 | 3UTR |
| hsa-miR-6742-3p | NM_173799 | 1816 | 1843 | 1 | 0.01332 | 0.38144 | 0.45372 | 27 | 3UTR |
| hsa-miR-6745 | NM_173799 | 1695 | 1730 | 1 | 0.01333 | 0.51413 | -0.02236 | 20 | 3UTR |
| hsa-miR-6746-3p | NM_173799 | 2692 | 2738 | 1 | 0.00021 | 0 | 0 | 46 | 3UTR |
| hsa-miR-6749-3p | NM_173799 | 1423 | 1465 | 1 | 0.00023 | 0.62616 | 0.37776 | 23 | 3UTR |
| hsa-miR-6750-3p | NM_173799 | 1834 | 1853 | 1 | 0.00205 | 0.28994 | 0.27661 | 19 | 3UTR |
| hsa-miR-6754-5p | NM_173799 | 2651 | 2696 | 1 | 0.00017 | 0 | 0 | 45 | 3UTR |
| hsa-miR-6757-3p | NM_173799 | 1436 | 1455 | 1 | 0.00151 | 0.02903 | 0.57599 | 19 | 3UTR |
| hsa-miR-6757-3p | NM_173799 | 1819 | 1843 | 1 | 0.01332 | 0.2395 | 0.26756 | 24 | 3UTR |
| hsa-miR-6758-5p | NM_173799 | 1958 | 2005 | 1 | 0.00015 | 0.43449 | 0.48972 | 20 | 3UTR |
| hsa-miR-6759-3p | NM_173799 | 2440 | 2475 | 1 | 0.00084 | 0 | 0 | 29 | 3UTR |
| hsa-miR-6760-5p | NM_173799 | 1967 | 2007 | 1 | 0.00131 | 0.41485 | 0.42525 | 18 | 3UTR |
| hsa-miR-6760-3p | NM_173799 | 1816 | 1844 | 1 | 0.01573 | 0.12569 | 0.30432 | 28 | 3UTR |
| hsa-miR-6762-3p | NM_173799 | 1498 | 1548 | 1 | 0.00018 | 0.30312 | 0.77422 | 22 | 3UTR |
| hsa-miR-6763-3p | NM_173799 | 2464 | 2483 | 1 | 0.01643 | 0 | 0 | 19 | 3UTR |
| hsa-miR-6767-5p | NM_173799 | 840 | 874 | 1 | 0.14926 | 2.30567 | 1.57609 | 23 | 3UTR |
| hsa-miR-6769a-3p | NM_173799 | 2010 | 2031 | 1 | 0.00036 | 0.19648 | 0.33998 | 21 | 3UTR |
| hsa-miR-6771-5p | NM_173799 | 1629 | 1649 | 1 | 0.00021 | 0.43914 | 0.96633 | 20 | 3UTR |
| hsa-miR-6771-3p | NM_173799 | 1834 | 1855 | 1 | 0.00211 | 0.36286 | 0.20007 | 21 | 3UTR |
| hsa-miR-6772-3p | NM_173799 | 1418 | 1472 | 1 | 0.00307 | 1.22888 | 0.34717 | 18 | 3UTR |
| hsa-miR-6773-3p | NM_173799 | 1816 | 1848 | 1 | 0.00124 | 0.24617 | 0.37266 | 18 | 3UTR |
| hsa-miR-6775-3p | NM_173799 | 1830 | 1855 | 1 | 0.00211 | 0.24 | 0.2686 | 25 | 3UTR |
| hsa-miR-6777-3p | NM_173799 | 1816 | 1852 | 1 | 0.00158 | 0.37273 | 0.41463 | 24 | 3UTR |
| hsa-miR-6777-3p | NM_173799 | 1454 | 1484 | 1 | 0.00089 | 0.2395 | 0.48177 | 30 | 3UTR |
| hsa-miR-6778-3p | NM_173799 | 2010 | 2028 | 1 | 0.00023 | 0.19417 | 0.35454 | 18 | 3UTR |
| hsa-miR-6779-5p | NM_173799 | 1619 | 1649 | 1 | 0.00021 | 0.40847 | 0.78156 | 23 | 3UTR |
| hsa-miR-6779-3p | NM_173799 | 1426 | 1474 | 1 | 0.00189 | 1.15585 | 0.35819 | 20 | 3UTR |
| hsa-miR-6780a-5p | NM_173799 | 831 | 846 | 1 | 5.29E-05 | 2.93496 | 1.46884 | 15 | 3UTR |
| hsa-miR-6781-3p | NM_173799 | 1443 | 1469 | 1 | 0.00097 | 0.21698 | 0.6127 | 26 | 3UTR |
| hsa-miR-6784-5p | NM_173799 | 1511 | 1533 | 1 | 0.00513 | 0.73562 | 0.73873 | 22 | 3UTR |
| hsa-miR-6784-3p | NM_173799 | 2445 | 2484 | 1 | 0.0042 | 0 | 0 | 21 | 3UTR |
| hsa-miR-6784-3p | NM_173799 | 1364 | 1391 | 1 | 0.00291 | 0.31504 | 0.29334 | 27 | 3UTR |
| hsa-miR-6785-5p | NM_173799 | 2702 | 2730 | 1 | 0.00017 | 0 | 0 | 28 | 3UTR |
| hsa-miR-6787-3p | NM_173799 | 1454 | 1484 | 1 | 0.00089 | 0.32923 | 0.63228 | 30 | 3UTR |
| hsa-miR-6788-5p | NM_173799 | 907 | 922 | 1 | 0.14384 | 2.16738 | 1.84856 | 15 | 3UTR |
| hsa-miR-6788-3p | NM_173799 | 2017 | 2043 | 1 | 0.07528 | -0.00918 | 0.3431 | 26 | 3UTR |
| hsa-miR-6789-3p | NM_173799 | 2683 | 2707 | 1 | 0.00077 | 0 | 0 | 24 | 3UTR |
| hsa-miR-6791-3p | NM_173799 | 1421 | 1441 | 1 | 0.00133 | 0.92859 | 0.41325 | 20 | 3UTR |
| hsa-miR-6792-3p | NM_173799 | 1806 | 1845 | 1 | 0.01627 | 0.17831 | 0.43998 | 17 | 3UTR |
| hsa-miR-6794-3p | NM_173799 | 2467 | 2490 | 1 | 0.00108 | 0 | 0 | 23 | 3UTR |
| hsa-miR-6796-3p | NM_173799 | 2017 | 2062 | 1 | 7.67E-05 | 0.35908 | 0.3899 | 45 | 3UTR |
| hsa-miR-6797-3p | NM_173799 | 1819 | 1844 | 1 | 0.01573 | 0.31034 | 0.3581 | 25 | 3UTR |
| hsa-miR-6798-3p | NM_173799 | 1803 | 1839 | 1 | 0.066 | 0.60658 | 0.48695 | 23 | 3UTR |
| hsa-miR-6800-3p | NM_173799 | 1436 | 1463 | 1 | 0.00252 | 0.17307 | 0.5919 | 27 | 3UTR |
| hsa-miR-6801-5p | NM_173799 | 2120 | 2143 | 1 | 0.0068 | 2.01277 | 0.91295 | 23 | 3UTR |
| hsa-miR-6801-3p | NM_173799 | 1832 | 1853 | 1 | 0.00205 | 0.50844 | 0.1934 | 21 | 3UTR |
| hsa-miR-6801-3p | NM_173799 | 1447 | 1472 | 1 | 0.00307 | 0.19405 | 0.56865 | 25 | 3UTR |
| hsa-miR-6805-3p | NM_173799 | 1816 | 1845 | 1 | 0.01627 | 0.10366 | 0.31381 | 29 | 3UTR |
| hsa-miR-6809-5p | NM_173799 | 918 | 940 | 1 | 0.10954 | 2.3989 | 1.70417 | 22 | 3UTR |
| hsa-miR-6811-5p | NM_173799 | 1621 | 1638 | 1 | 1.14E-05 | 0.92661 | 0.83051 | 17 | 3UTR |
| hsa-miR-6817-5p | NM_173799 | 1575 | 1596 | 1 | 0.0068 | 1.84326 | 0.93084 | 21 | 3UTR |
| hsa-miR-6818-3p | NM_173799 | 1423 | 1471 | 1 | 0.00351 | 1.43049 | 0.37165 | 18 | 3UTR |
| hsa-miR-6819-5p | NM_173799 | 906 | 956 | 1 | 0.2124 | 2.01845 | 1.60139 | 28 | 3UTR |
| hsa-miR-6819-3p | NM_173799 | 1379 | 1407 | 1 | 0.00153 | 0.773 | 0.17709 | 28 | 3UTR |
| hsa-miR-6821-3p | NM_173799 | 2004 | 2029 | 1 | 0.00025 | 0.0797 | 0.35974 | 15 | 3UTR |
| hsa-miR-6822-3p | NM_173799 | 1655 | 1700 | 1 | 0.00032 | 0.16608 | 0.43405 | 45 | 3UTR |
| hsa-miR-6828-3p | NM_173799 | 1423 | 1468 | 1 | 0.00118 | 0.56876 | 0.62494 | 45 | 3UTR |
| hsa-miR-6829-5p | NM_173799 | 1220 | 1237 | 1 | 0.00108 | -0.5409 | 0.76565 | 17 | 3UTR |
| hsa-miR-6830-5p | NM_173799 | 2259 | 2290 | 1 | 5.49E-05 | 0 | 0 | 26 | 3UTR |
| hsa-miR-6830-3p | NM_173799 | 1331 | 1351 | 1 | 0.18241 | -0.4974 | 1.11806 | 20 | 3UTR |
| hsa-miR-6832-3p | NM_173799 | 1789 | 1832 | 1 | 0.00067 | 0.27572 | 0.35555 | 27 | 3UTR |
| hsa-miR-6833-5p | NM_173799 | 912 | 928 | 1 | 0.37618 | 2.3066 | 1.77637 | 16 | 3UTR |
| hsa-miR-6841-3p | NM_173799 | 1436 | 1463 | 1 | 0.00252 | 1.23063 | 0.43283 | 27 | 3UTR |
| hsa-miR-6842-3p | NM_173799 | 1423 | 1456 | 1 | 0.00121 | 0.65553 | 0.59312 | 33 | 3UTR |
| hsa-miR-6847-5p | NM_173799 | 2273 | 2289 | 1 | 5.29E-05 | 0 | 0 | 16 | 3UTR |
| hsa-miR-6856-5p | NM_173799 | 1134 | 1164 | 1 | 0.01547 | 0.78139 | 1.17801 | 30 | 3UTR |
| hsa-miR-6856-3p | NM_173799 | 1816 | 1838 | 1 | 0.05672 | 0.60658 | 0.49356 | 22 | 3UTR |
| hsa-miR-6769b-3p | NM_173799 | 1831 | 1852 | 1 | 0.00158 | 0.23134 | 0.26523 | 21 | 3UTR |
| hsa-miR-6865-5p | NM_173799 | 906 | 926 | 1 | 0.28926 | 1.42359 | 1.73354 | 20 | 3UTR |
| hsa-miR-6866-5p | NM_173799 | 1207 | 1240 | 1 | 0.00162 | 0.54676 | 0.83785 | 22 | 3UTR |
| hsa-miR-6866-3p | NM_173799 | 1836 | 1855 | 1 | 0.00211 | 0.45298 | 0.22399 | 19 | 3UTR |
| hsa-miR-6870-5p | NM_173799 | 1992 | 2009 | 1 | 0.00086 | 0.42005 | 0.41485 | 17 | 3UTR |
| hsa-miR-6870-3p | NM_173799 | 1414 | 1466 | 1 | 0.00031 | 0.5206 | 0.68857 | 44 | 3UTR |
| hsa-miR-6873-3p | NM_173799 | 1834 | 1852 | 1 | 0.00158 | 0.45298 | 0.22399 | 18 | 3UTR |
| hsa-miR-6876-3p | NM_173799 | 1338 | 1360 | 1 | 0.00444 | -0.34668 | 1.20616 | 22 | 3UTR |
| hsa-miR-6878-3p | NM_173799 | 1816 | 1839 | 1 | 0.066 | 0.59679 | 0.51111 | 23 | 3UTR |
| hsa-miR-6879-5p | NM_173799 | 912 | 941 | 1 | 0.10647 | 2.25847 | 1.68337 | 29 | 3UTR |
| hsa-miR-6879-5p | NM_173799 | 1990 | 2008 | 1 | 0.00114 | 0.61243 | 0.35766 | 18 | 3UTR |
| hsa-miR-6879-5p | NM_173799 | 1717 | 1737 | 1 | 0.19041 | -0.05068 | 0.45852 | 20 | 3UTR |
| hsa-miR-6880-3p | NM_173799 | 1829 | 1853 | 1 | 0.00205 | 0.25025 | 0.27416 | 24 | 3UTR |
| hsa-miR-6883-3p | NM_173799 | 1830 | 1852 | 1 | 0.00158 | 0.10173 | 0.27588 | 22 | 3UTR |
| hsa-miR-6884-3p | NM_173799 | 2009 | 2031 | 1 | 0.00036 | 0.19417 | 0.35454 | 22 | 3UTR |
| hsa-miR-6885-3p | NM_173799 | 1454 | 1471 | 1 | 0.00351 | 0.20891 | 0.56253 | 17 | 3UTR |
| hsa-miR-6886-3p | NM_173799 | 2469 | 2491 | 1 | 0.00099 | 0 | 0 | 22 | 3UTR |
| hsa-miR-6887-3p | NM_173799 | 1816 | 1835 | 1 | 0.03282 | 0.0845 | 0.3398 | 19 | 3UTR |
| hsa-miR-6888-5p | NM_173799 | 1039 | 1063 | 1 | 0.0293 | 0.967 | 1.31017 | 24 | 3UTR |
| hsa-miR-6891-3p | NM_173799 | 1805 | 1834 | 1 | 0.02077 | 0.26886 | 0.44902 | 29 | 3UTR |
| hsa-miR-6891-3p | NM_173799 | 1425 | 1450 | 1 | 0.00458 | 0.91657 | 0.3435 | 16 | 3UTR |
| hsa-miR-6895-3p | NM_173799 | 947 | 989 | 1 | 0.0007 | 2.78463 | 1.84122 | 21 | 3UTR |
| hsa-miR-7107-3p | NM_173799 | 1420 | 1453 | 1 | 0.00534 | 1.00902 | 0.44874 | 33 | 3UTR |
| hsa-miR-7110-3p | NM_173799 | 2017 | 2032 | 1 | 0.00043 | 0.15905 | 0.35974 | 15 | 3UTR |
| hsa-miR-7112-5p | NM_173799 | 2119 | 2134 | 1 | 0.00063 | 1.92265 | 0.89319 | 15 | 3UTR |
| hsa-miR-7151-5p | NM_173799 | 2454 | 2478 | 1 | 0.00043 | 0 | 0 | 24 | 3UTR |
| hsa-miR-7152-5p | NM_173799 | 1420 | 1471 | 1 | 0.00351 | 1.47128 | 0.36186 | 20 | 3UTR |
| hsa-miR-7160-5p | NM_173799 | 1958 | 1979 | 1 | 0.00092 | 0.31937 | 0.49804 | 21 | 3UTR |
| hsa-miR-7160-3p | NM_173799 | 1914 | 1937 | 1 | 2.18E-07 | 0.20896 | 0.35142 | 23 | 3UTR |
| hsa-miR-7702 | NM_173799 | 1423 | 1460 | 1 | 0.00145 | 1.01446 | 0.50502 | 25 | 3UTR |
| hsa-miR-7702 | NM_173799 | 1828 | 1848 | 1 | 0.00124 | 0.0845 | 0.3398 | 20 | 3UTR |
| hsa-miR-7703 | NM_173799 | 1453 | 1472 | 1 | 0.00307 | 0.23727 | 0.59924 | 19 | 3UTR |
| hsa-miR-7843-5p | NM_173799 | 1240 | 1281 | 1 | 3.51E-05 | 0.91004 | 1.23185 | 41 | 3UTR |
| hsa-miR-1273h-5p | NM_173799 | 1709 | 1730 | 1 | 0.01333 | -0.0036 | 0.33004 | 21 | 3UTR |
| hsa-miR-1273h-3p | NM_173799 | 1421 | 1468 | 1 | 0.00118 | 0.8223 | 0.59068 | 47 | 3UTR |
| hsa-miR-7846-3p | NM_173799 | 2257 | 2279 | 1 | 0.00607 | 0 | 0 | 17 | 3UTR |
| hsa-miR-7848-3p | NM_173799 | 958 | 982 | 1 | 0.00031 | 0.86354 | 1.95134 | 17 | 3UTR |
| hsa-miR-7976 | NM_173799 | 1461 | 1483 | 1 | 0.00047 | 0.3199 | 0.55274 | 22 | 3UTR |
| hsa-miR-7977 | NM_173799 | 1915 | 1931 | 1 | 1.65E-06 | 0.16529 | 0.42525 | 16 | 3UTR |
| hsa-miR-7977 | NM_173799 | 1829 | 1842 | 1 | 0.01144 | 0.03556 | 0.26664 | 13 | 3UTR |
| hsa-miR-8056 | NM_173799 | 1957 | 1998 | 1 | 0.00171 | 0.31937 | 0.46373 | 41 | 3UTR |
| hsa-miR-8056 | NM_173799 | 1707 | 1728 | 1 | 0.01105 | 0.18076 | 0.20768 | 21 | 3UTR |
| hsa-miR-8057 | NM_173799 | 1142 | 1156 | 1 | 0.00017 | 0.78354 | 1.24287 | 14 | 3UTR |
| hsa-miR-8059 | NM_173799 | 936 | 955 | 1 | 0.20268 | 2.41754 | 1.69561 | 19 | 3UTR |
| hsa-miR-8063 | NM_173799 | 1522 | 1536 | 1 | 0.00397 | 0.64574 | 0.73751 | 14 | 3UTR |
| hsa-miR-8069 | NM_173799 | 1137 | 1158 | 1 | 0.00179 | 2.03577 | 1.10093 | 21 | 3UTR |
| hsa-miR-8485 | NM_173799 | 850 | 875 | 1 | 0.16796 | 1.09603 | 1.89477 | 22 | 3UTR |
| hsa-miR-9718 | NM_173799 | 1524 | 1550 | 1 | 0.00071 | 0.85131 | 0.86477 | 26 | 3UTR |
| hsa-miR-9898 | NM_173799 | 1819 | 1844 | 1 | 0.01573 | -0.07165 | 0.33876 | 25 | 3UTR |
| hsa-miR-9986 | NM_173799 | 1570 | 1610 | 1 | 0.0171 | 1.43559 | 0.64207 | 40 | 3UTR |
| hsa-miR-10398-3p | NM_173799 | 1527 | 1573 | 1 | 0.00791 | 0.88394 | 1.22451 | 46 | 3UTR |
| hsa-miR-10399-3p | NM_173799 | 1439 | 1462 | 1 | 0.00252 | 0.02468 | 0.58211 | 23 | 3UTR |
| hsa-miR-10401-3p | NM_173799 | 1419 | 1450 | 1 | 0.00458 | 0.71671 | 0.47443 | 31 | 3UTR |
| hsa-miR-11181-5p | NM_173799 | 2435 | 2479 | 1 | 0.00062 | 0 | 0 | 33 | 3UTR |
| hsa-miR-3059-3p | NM_173799 | 1993 | 2013 | 1 | 0.00027 | 0.89111 | 0.25367 | 20 | 3UTR |
| hsa-miR-3059-3p | NM_173799 | 1810 | 1824 | 1 | 1.43E-05 | 0.08217 | 0.4516 | 14 | 3UTR |
| hsa-miR-3085-3p | NM_173799 | 1828 | 1849 | 1 | 0.00075 | -0.11535 | 0.32213 | 21 | 3UTR |
| hsa-miR-6529-3p | NM_173799 | 1379 | 1404 | 1 | 0.00053 | 0.5454 | 0.0792 | 19 | 3UTR |
| hsa-miR-12116 | NM_173799 | 2009 | 2029 | 1 | 0.00025 | 0.36766 | 0.32438 | 20 | 3UTR |
| hsa-miR-12122 | NM_173799 | 1636 | 1659 | 1 | 1.18E-05 | 0.01548 | 0.7473 | 23 | 3UTR |
| hsa-miR-12124 | NM_173799 | 915 | 936 | 1 | 0.15048 | 1.96072 | 1.74333 | 21 | 3UTR |
